# Hormonal pleiotropy structures genetic covariance

**DOI:** 10.1002/evl3.240

**Published:** 2021-06-13

**Authors:** Tyler N. Wittman, Christopher D. Robinson, Joel W. McGlothlin, Robert M. Cox

**Affiliations:** ^1^ Department of Biology University of Virginia Charlottesville Virginia 22904; ^2^ Department of Biological Sciences Virginia Tech Blacksburg Virginia 24061

**Keywords:** Animal model, *Anolis*, **B** matrix, **G** matrix, genetic correlation, intralocus sexual conflict, quantitative genetics, sexual dimorphism, testosterone

## Abstract

Quantitative genetic theory proposes that phenotypic evolution is shaped by **G**, the matrix of genetic variances and covariances among traits. In species with separate sexes, the evolution of sexual dimorphism is also shaped by **B**, the matrix of between‐sex genetic variances and covariances. Despite considerable focus on estimating these matrices, their underlying biological mechanisms are largely speculative. We experimentally tested the hypothesis that **G** and **B** are structured by hormonal pleiotropy, which occurs when one hormone influences multiple phenotypes. Using juvenile brown anole lizards (*Anolis sagrei*) bred in a paternal half‐sibling design, we elevated the steroid hormone testosterone with slow‐release implants while administering empty implants to siblings as a control. We quantified the effects of this manipulation on the genetic architecture of a suite of sexually dimorphic traits, including body size (males are larger than females) and the area, hue, saturation, and brightness of the dewlap (a colorful ornament that is larger in males than in females). Testosterone masculinized females by increasing body size and dewlap area, hue, and saturation, while reducing dewlap brightness. Control females and males differed significantly in **G**, but treatment of females with testosterone rendered **G** statistically indistinguishable from males. Whereas **B** was characterized by low between‐sex genetic correlations when estimated between control females and males, these same correlations increased significantly when estimated between testosterone females and either control or testosterone males. The full **G** matrix (including **B**) for testosterone females and either control or testosterone males was significantly less permissive of sexually dimorphic evolution than was **G** estimated between control females and males, suggesting that natural sex differences in testosterone help decouple genetic variance between the sexes. Our results confirm that hormonal pleiotropy structures genetic covariance, implying that hormones play an important yet overlooked role in mediating evolutionary responses to selection.

**Impact Summary**

Quantitative genetics was originally developed by plant and animal breeders as a way to predict how crops and livestock would respond to artificial selection, then subsequently adopted by evolutionary biologists interested in natural selection. In both cases, quantitative geneticists have shown that evolutionary change can be accurately predicted using simple statistical estimates of the genetic variances and covariances of traits. However, the simplicity of statistical quantitative genetics tends to obscure the underlying biology of how and why traits exhibit particular patterns of genetic variance and covariance. We provide the first experimental demonstration that, by simultaneously influencing the expression of multiple traits, hormones structure quantitative genetic variance and covariance. Specifically, we show that a single hormone (testosterone) can alter the genetic variances and covariances associated with a suite of traits (body size and the size and color of a sexual ornament) in a lizard. Although females and males of this species share the vast majority of their genome, they nonetheless exhibit distinct patterns of genetic variance and covariance for these traits. However, in the presence of testosterone, females exhibit patterns of genetic variance and covariance that are similar to those of males. Though it is well known that testosterone regulates growth and ornamentation in many species, ours is the first study to show that this hormone also shapes the underlying genetic parameters that determine how a population will respond to selection. In addition to providing a biological explanation for the mathematical parameters of quantitative genetics, our results also illustrate how fundamental sex differences in circulating levels of a single hormone can facilitate the independent evolution of females and males despite the constraint of a shared genome.

## Introduction

When natural selection acts on phenotypes, the evolutionary response of a population depends on the extent to which these phenotypes are heritable and genetically correlated with one another. In quantitative genetics, these properties are often represented by the genetic variance‐covariance matrix, **G** (Lande 1979; Lande and Arnold 1983; Eqns. 1–2, Supporting Information). In addition to its importance for evolutionary theory (Steppan et al. 2002; Jones et al. 2007; Roff 2007), **G** can inform studies of adaptation and reveal properties of the genotype‐phenotype map (Grant and Grant 1995; Wilson et al. 2010; Milocco and Salazar‐Ciudad 2020). Accordingly, estimates of **G** have been obtained for a variety of species (Arnold et al. 2008; Pitchers et al. 2014; Wood and Brodie 2015), comparative studies have explored its evolution (Chenoweth et al. 2010; McGlothlin et al. 2018; Walter et al. 2018), and experiments have characterized its sensitivity to the environment (Sgro and Hoffmann 2004; Charmantier and Garant 2005; Wood and Brodie 2015) and to mutation (Camara and Pigliucci 1999; Estes et al. 2005). By contrast, no experiment has explored how **G** is structured by internal physiological mechanisms that mediate the translation of genotype to phenotype, such as hormones.

In species with separate sexes, phenotypic evolution also depends on patterns of genetic covariance between females and males, as represented by the sub‐matrix **B** within **G** (Lande 1980; Eqn. 3, Supporting Information). Between‐sex genetic covariance represents a short‐term constraint on the evolution of sexual dimorphism, but it is also predicted to break down over time in response to sexually antagonistic selection (Lande 1980, 1987; Fairbairn and Roff 2006). Sexually antagonistic selection may not always reduce between‐sex covariance in the short term (McGlothlin et al. 2019), but selection experiments confirm that it can do so rapidly in some circumstances (Delph et al. 2011), and comparative studies indicate that the evolution of sexual dimorphism is generally associated with a reduction in between‐sex genetic covariance (Poissant et al. 2010). Although recent work has emphasized the importance of **B** in shaping the evolution of sexual dimorphism (Gosden et al. 2012; Wyman et al. 2013; Cheng and Houle 2020) and studies on a variety of species have empirically characterized **B** (Steven et al. 2007; Campbell et al. 2011; Lewis et al. 2011; Ingleby et al. 2014; Cox et al. 2017a; White et al. 2019), we know relatively little about the physiological mechanisms that orchestrate the breakdown of between‐sex genetic covariance to facilitate the evolution of sexual dimorphism (Cox et al. 2017b).

In this study, we experimentally test the hypothesis that hormonal pleiotropy structures **G** and **B**. Hormonal pleiotropy (one hormone influencing multiple phenotypes) is analogous to genetic pleiotropy (one gene influencing multiple phenotypes) with the substitution of a hormone and its receptor in place of a gene in the literal sense (Ketterson and Nolan 1999; Lema 2014; Cox 2020). Hormonal pleiotropy has served as an important conceptual framework for evolutionary biology (Finch and Rose 1995; Flatt et al. 2005; Bourg et al. 2019), but only a handful of studies have formally integrated this concept with quantitative genetics (McGlothlin and Ketterson 2008; Ketterson et al. 2009; Cox et al. 2016; Dantzer and Swanson 2017; Cox 2020). To test whether hormonal pleiotropy structures **G** and **B**, we focus on the steroid hormone testosterone, which naturally circulates at higher levels in adult males than in females. In vertebrate genomes, hundreds to thousands of genes contain response elements that bind the androgen receptor, such that testosterone can exert massively pleiotropic effects (reviewed by Cox 2020). Consequently, sex differences in circulating testosterone lead to sex differences in the transcription and translation of shared genes into dimorphic phenotypes, which is predicted to produce sex‐specific patterns in **G** and break down between‐sex covariance in **B**.

We test these predictions in the brown anole (*Anolis sagrei*), a sexually dimorphic lizard in which males are larger than females and possess a large and colorful ornament (dewlap) that is much smaller in females (Cox and Calsbeek 2010; Cox et al. 2017a). These sex differences are regulated in part by maturational divergence in testosterone, which enhances growth and dewlap development when administered to juveniles and restores these phenotypes in castrated adult males (Cox et al. 2009a; 2009b; 2015). Testosterone also alters the female transcriptome in ways that parallel natural sex differences in gene expression that emerge during maturation (Cox et al. 2016; 2017b; Cox 2020). Females and males differ in **G** for dewlap phenotypes, most of which are also characterized by relatively weak between‐sex genetic covariance in **B** (Cox et al. 2017a). Between‐sex genetic covariance for body size is high during early ontogeny, but it breaks down as sexual dimorphism develops, coincident with maturational increases in testosterone and sex‐biased gene expression (Cox et al. 2017b). Collectively, these studies suggest that females and males share a similar genetic architecture for body size and dewlap morphology, from which the sex‐specific expression of genetic variance and covariance is coordinated by maturational divergence in testosterone (Cox 2020). We provide the first experimental support for this hypothesis, and for the more general hypothesis that hormonal pleiotropy structures genetic covariance, by demonstrating pronounced changes in **G** and **B** in response to testosterone manipulation in a pedigreed breeding population of anoles.

## Materials and Methods

### BREEDING AND EXPERIMENTAL DESIGN

We bred anoles in a paternal half‐sibling design with two dams per sire (*n* = 120 dams, 60 sires) following published protocols (Cox et al. 2016; Cox et al. 2017a,2017b; Logan et al. 2018; see Supporting Information). Sample sizes and family sizes are summarized in Table S1. Dams and sires were F_2_ descendants of stock from Great Exuma in the Commonwealth of the Bahamas (23°29’N, 75°45’W; imported under permits from the Bahamas Environment, Science and Technology Commission, the Bahamas Ministry of Agriculture, and the United States Fish and Wildlife Service). Breeding was conducted in captivity with all F_1_ and F_2_ crosses set to avoid inbreeding. All procedures were reviewed and approved by the University of Virginia's Animal Care and Use Committee (protocol 3896).

We raised F_3_ progeny to 3 months of age and then administered one of two treatments: (1) a slow‐release implant containing 100 μg testosterone, or (2) an empty implant as a control. Implant design and surgical procedures followed previous studies (Cox et al. 2015; Cox et al. 2017b; see Supporting Information), in which identical implants elevated testosterone levels of juvenile males and females approximately 5‐fold relative to controls, while remaining within the natural physiological range for adult males. Because anoles lay a single egg every 7–14 days, progeny were produced continuously over 10 months (August 2017 to June 2018). To balance treatments within maternal families, we haphazardly determined whether the first offspring of each sex would receive a testosterone or a control implant for a given family, then alternated between treatment groups for all subsequent progeny of each sex. At 8 months of age, we measured each individual for snout‐vent length (SVL) and photographed its dewlap to measure area, hue, saturation, and brightness following Cox et al. (2017a; see Supporting Information). We used these five traits to estimate **G** and **B**. Dewlap area and SVL are metric traits in which variance increases with the mean, so we ln‐transformed these traits, rendering values proportional and preventing sex and treatment differences in size from influencing total genetic variance.

### ESTIMATION OF GENETIC COVARIANCE MATRICES

We estimated **G** using the program WOMBAT (Meyer 2007) and a restricted error maximum likelihood (REML, animal model, see Supporting Information) framework that allowed us to incorporate three generations of pedigree information describing relationships among F_1_ grandparents, F_2_ parents, and F_3_ experimental progeny. We estimated separate within‐sex **G** matrices for each of the four experimental groups (control females, control males, testosterone females, testosterone males). For pairs of male and female treatments, we estimated full **G** matrices including both within‐sex matrices (**G_F_**, **G_M_**) and the between‐sex matrix (**B**). When estimated for control females and control males, **B** describes natural patterns of between‐sex covariance. When estimated for testosterone females and either control or testosterone males, **B** describes experimentally induced patterns of between‐sex covariance that occur when both sexes translate genotype to phenotype in the presence of testosterone. For all estimates, we included the month of hatching as a random effect to account for any inadvertent shifts in husbandry (e.g., size and number of crickets fed per individual) that may have occurred despite our best efforts at standardization. Inclusion of Dam ID as a random (maternal) effect did not significantly improve fit for any model, so it was not included in our final matrix estimates. We estimated **G** and **B** using penalized estimation with shrinkage of genetic partial autocorrelations toward zero by setting a mild penalty (sample size of beta distribution = 3.0) using the PACORR function in WOMBAT (Meyer 2011, 2016). To confirm significant genetic variance and covariance, we used likelihood ratio tests to compare models estimating the full **G** for each group (or the full **G** and **B** for each combination of female and male groups) against simpler models setting genetic covariances to zero or excluding additive genetic effects entirely (see Supporting Information).

In addition to **G**, we used WOMBAT to estimate phenotypic covariance matrices (**P**) describing overall patterns of trait variance and covariance across individuals without taking genetic relationships into account. We also used WOMBAT to estimate both phenotypic and genetic correlation matrices. Prior to analysis, we variance‐standardized our estimates of **G** and **B** by dividing genetic variances by phenotypic variances (narrow‐sense heritability, *h^2^*) and dividing genetic covariances by mean phenotypic variances (Hansen and Houle 2008). This standardization ensures that traits measured on different phenotypic scales (e.g., mm, degrees, percentages) can still contribute equal genetic variance to the matrix. Unstandardized matrices gave qualitatively identical results when compared among groups (see Supporting Information).

### STATISTICAL ANALYES AND MATRIX COMPARISONS

All statistical analyses were performed in R 3.5.3 (R Core Team 2019). To test for phenotypic effects of sex and testosterone, we individually analyzed each phenotype as the dependent variable in a linear mixed‐effects model with sex and treatment as fixed effects with interaction, plus the month of hatching, sire, and dam (nested within sire) as random effects. We conducted these analyses at 3 months of age to describe patterns of sexual dimorphism just prior to treatment, and at 8 months of age to describe the development of sexual dimorphism and the effects of testosterone. To clarify statistical interactions, we conducted similar analyses of treatment effects within each sex, as well as analyses of sex effects within each treatment. For analyses within each sex, we included ln SVL as a covariate to assess treatment effects on dewlap phenotypes independent of effects on size. We also conducted PCA analyses to compare multivariate sex and treatment effects in reduced phenotypic space (Supporting Information).

To test whether testosterone shapes **G**, we conducted pairwise matrix comparisons between all experimental groups using random skewers (Cheverud 1996; Cheverud and Marroig 2007). We generated 10,000 random skewers by drawing each gradient in each vector from a normal distribution with a mean of 0 and standard deviation of 1 (Marroig et al. 2011), then standardizing each vector to a norm of 1. These vectors represent **β** in Eqn. 2 (Supporting Information). We multiplied each skewer by each **G** matrix to derive 10,000 vectors of evolutionary response for each matrix ($\Delta \bar{z}$ in Eqn. 2), then calculated the mean correlation between response vectors as an estimate of similarity between any two matrices. If testosterone structures **G**, the matrix of control females should exhibit low correlations with those of all other groups, and treatment of females with testosterone should produce a matrix more highly correlated with those of males. In the hypothetical absence of sex differences and treatment effects, the null hypothesis is that each estimate of **G** should be identical (*r* = 1) aside from sampling error. To test for sex and treatment effects while incorporating error in **G**, we simulated a sampling distribution for each matrix using the REML‐MVN method in WOMBAT (Meyer and Houle 2013; Houle and Meyer 2015), with 10,000 samples per matrix. We used random skewers to produce a null distribution of 10,000 mean vector correlations between our best estimate of **G** from each group and each of the 10,000 simulated matrices in its own sampling distribution. This null distribution describes how correlated each matrix is expected to be with itself, given sampling error. We then asked whether the best estimate of **G** from each of the other experimental groups produced a mean vector correlation that fell below the lower 5% bound of this null distribution when compared to the best estimate of **G** from the reference group. We compared correlation matrices using modified versions of the Mantel test and the T method (see Supporting Information).

To test whether natural sex differences in testosterone contribute to the breakdown of between‐sex genetic correlations, we estimated full **G** matrices (including **B**) for (1) control females and control males, (2) testosterone females and control males, and (3) testosterone females and testosterone males. We then converted the five diagonal elements in **B** to genetic correlations (*r*_MF_) and used paired (by trait) *t*‐tests to assess whether these correlations are weaker in the correlation matrix for control females and control males than in either of the matrices including testosterone females. To incorporate uncertainty in matrix estimation, we also obtained *r*_MF_ values for each of the simulations in the REML‐MVN error distribution for each matrix and calculated the mean difference in *r*_MF_ values (paired by trait) between 10,000 pairs of matrices from each distribution. We then tested whether the lower 5% bound of this distribution fell above zero when subtracting *r*_MF_ values in the control female and control male matrix from *r*_MF_ values in either of the matrices including testosterone females.

To test whether natural sex differences in testosterone structure both **G** and **B** in ways that could potentially influence the evolution of sexual dimorphism, we compared the full **G** matrices (including **G_F_**, **G_M_** and **B**) using sexually antagonistic skewers (Cox et al. 2017a). In this modification of random skewers, the magnitude of each selection gradient is drawn from a normal distribution and vectors are standardized to a norm of 1, but gradients for each homologous trait are constrained to be opposite in sign between sexes. We passed 10,000 sexually antagonistic skewers through each matrix and calculated the mean vector correlations between response vectors of (1) control females and control males, (2) testosterone females and control males, and (3) testosterone females and testosterone males. We compared these mean vector correlations to null distributions created by applying the same sexually antagonistic skewers to each of the 10,000 simulated matrices in the REML‐MVN distribution for each matrix. Our *a priori* prediction was that natural sex differences in testosterone shape **G** and **B** in ways that should facilitate the evolution of sexual dimorphism, such that the mean vector correlation between responses of testosterone females and either control males or testosterone males should be higher than that between control females and control males. Therefore, we tested whether the mean vector correlation for testosterone females and either male group fell above the upper 5% bound of the simulated distribution for control females and control males, and whether the mean vector correlation for control females and control males fell below the lower 5% bound of the simulated distribution for testosterone females and either male group.

## Results and Discussion

### SEXUAL DIMORPHISM AND PHENOTYPIC EFFECTS OF TESTOSTERONE

**Figure 1:**
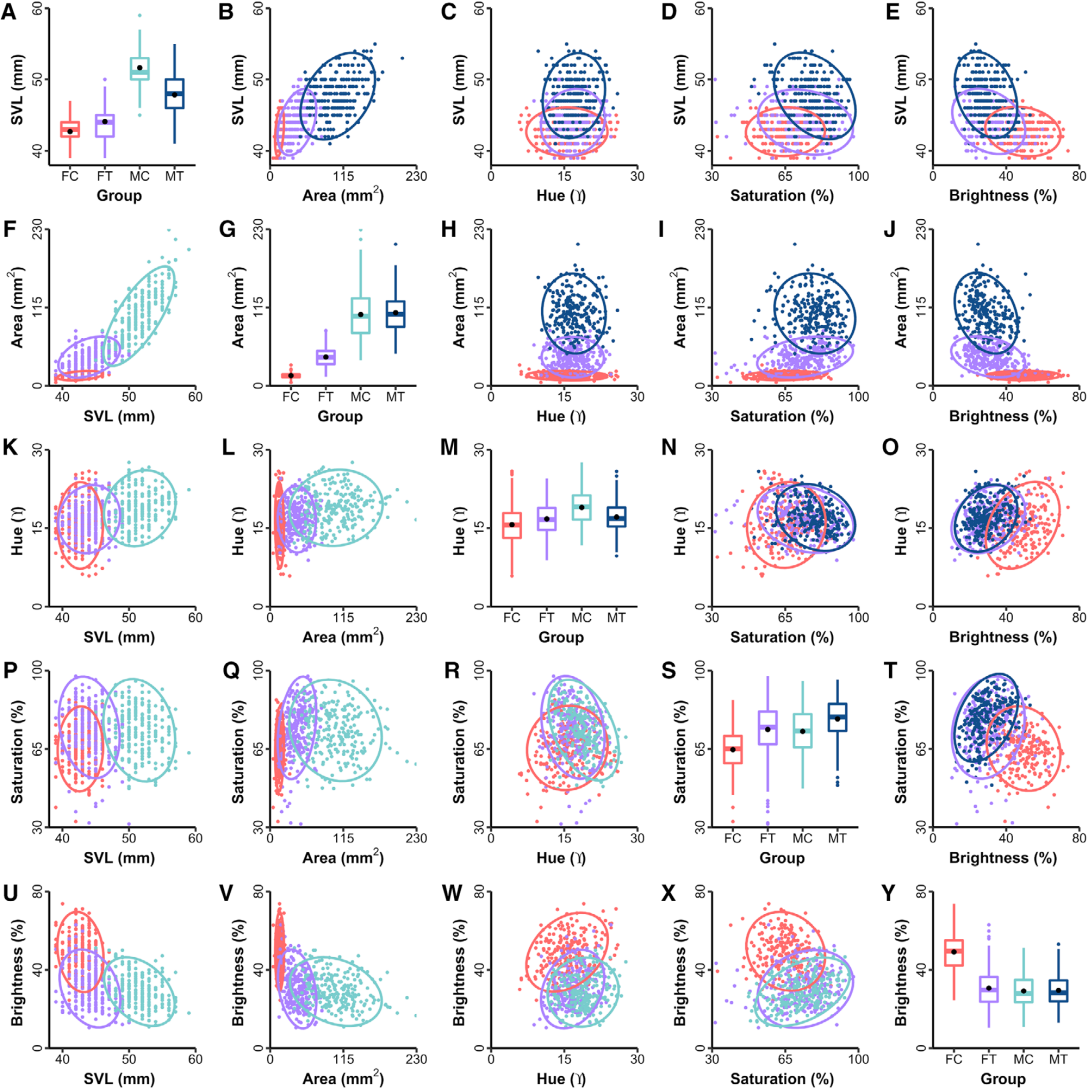
Effects of sex and testosterone treatment on phenotypic means, variances, and covariances for five traits. Panels on the diagonal show the raw phenotypic distributions (dot = mean, bar = median, box = inter‐quartiles, whiskers = 95% CIs) for each of four experimental groups (FC = control female, FT = testosterone female, MC = control male, MT = testosterone male). Panels above or below the diagonal show bivariate relationships between trait pairs with covariance ellipses corresponding to 95% CIs. For ease of visual comparison, control males are only plotted below the diagonal and testosterone males are only plotted above the diagonal.

At 3 months of age (pre‐treatment), sex differences were absent for dewlap hue, minor for dewlap brightness, and pronounced for SVL, dewlap area, and dewlap saturation (Table S2). There was no initial difference in any phenotype with respect to the treatments that were subsequently assigned (Table S2). By 8 months of age (post‐treatment), control females and males had diverged substantially in all phenotypes, but sexual dimorphism was reduced (for SVL, dewlap size, and dewlap saturation) or absent (for dewlap hue and dewlap brightness) between testosterone females and males (Fig. 1, Table S3). Treatment of females with testosterone increased SVL, dewlap area, dewlap saturation, and dewlap hue while decreasing dewlap brightness (Fig. 1, Fig. S1; Table S4). PC1 explained 49% of phenotypic variance and clearly separated control females from both male groups, with testosterone females intermediate (Fig. S2). These sex differences and treatment effects are broadly consistent with previous studies (Cox et al. 2015; 2016; 2017a,2017b) and confirm that subsequent comparisons of **G** and **B** involve a suite of traits that were sexually dimorphic and responsive to testosterone.

### TESTOSTERONE STRUCTURES GENETIC COVARIANCE

**Figure 2:**
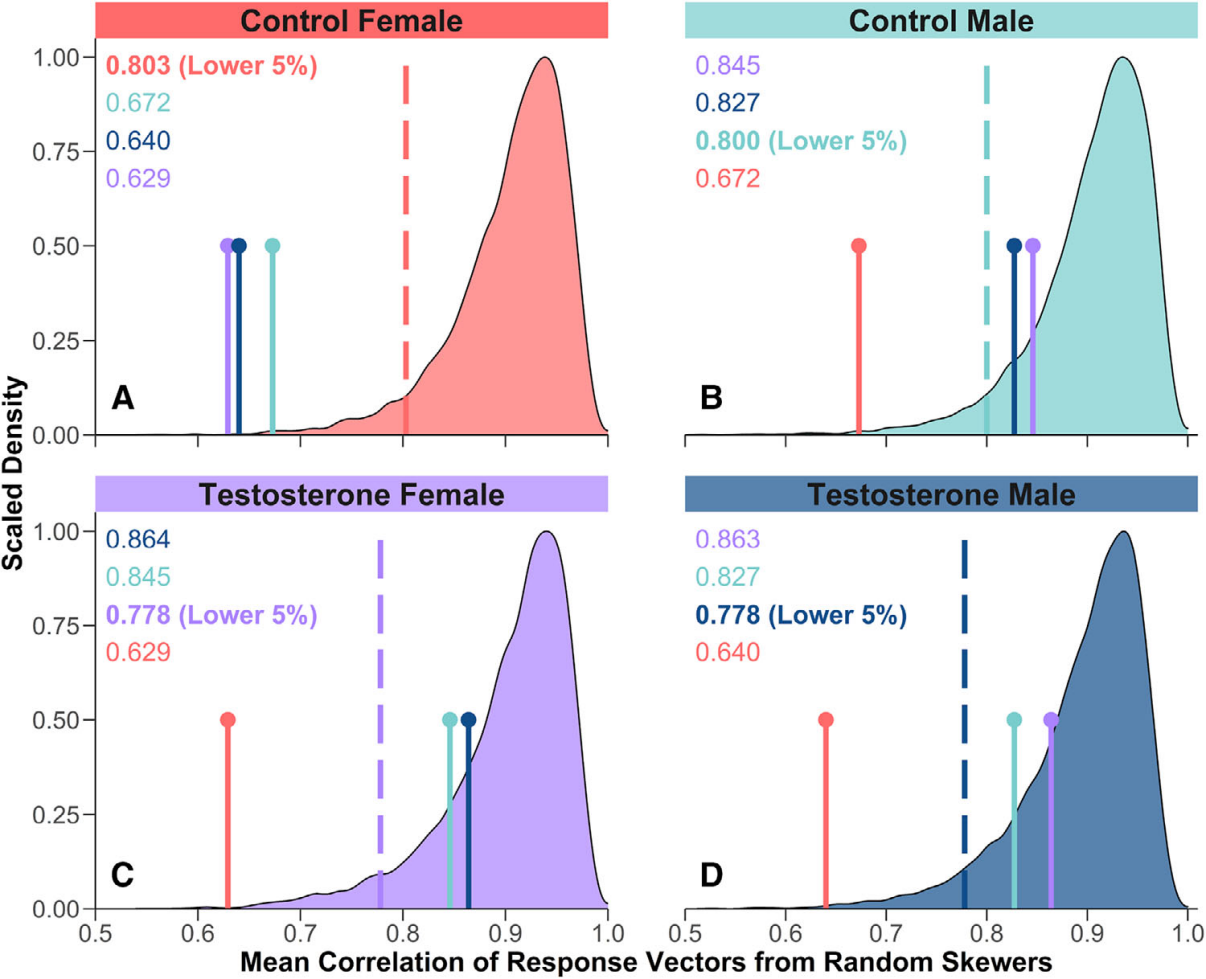
Comparisons of within‐sex **G** matrices across four experimental groups based on predicted evolutionary responses to random skewers. The null distribution of mean response vector correlations between the best estimate of **G** for a group and each of the 10,000 simulated matrices from its own sampling distribution is shown separately for (A) control females, (B) control males, (C) testosterone females, and (D) testosterone males. Dashed lines indicate the lower 5% bound of each distribution. Vertical pins indicate mean vector correlations between the best estimate of **G** for each of three comparison groups to that of the reference group whose null distribution is shown in that panel. Note that each vector correlation is plotted on two panels to facilitate comparison to each of the corresponding null distributions.

In each experimental group, the full **G** matrix was preferred over simpler models excluding additive genetic (co)variance (Table S5). Best estimates of **G**, **P**, and associated correlation matrices (see Tables S6‐S9) were always statistically distinct between control females and control males (Fig. 2; Fig. S3; Tables S10‐S11). However, random skewers analyses revealed that testosterone shifted **G** of females toward a genetic architecture similar to that of males (Fig. 2; Table S10). The mean vector correlation between evolutionary responses was low for control females when compared to control males (*r* = 0.67), testosterone males (*r* = 0.64), and testosterone females (*r* = 0.63). All three of these correlations fell outside the lower 5% bounds of the error distributions for each individual matrix (Fig. 2). By contrast, the mean vector correlation was high between testosterone females and either control males (*r* = 0.85) or testosterone males (*r* = 0.86), similar to the expectedly high correlation between control and testosterone males (*r* = 0.83). None of these three correlations fell outside the lower 5% bounds of the matrices being compared (Fig. 2). The same patterns of statistical separation were observed when using random skewers to compare unstandardized **G** matrices and when using several additional methods to compare genetic correlation matrices (Table S10). Therefore, elevating testosterone in females significantly altered **G**, producing a matrix that was statistically indistinguishable from that of males. Presumably, this occurred because some patterns of genetic variance and covariance that are naturally present in females were masked by the overriding “environmental” effect of elevated testosterone, whereas other patterns that are naturally “cryptic” in females were revealed via activation of underlying genes by testosterone. Consistent with this second idea, additive genetic variance for SVL increases as male anoles mature, coincident with the transcriptional activation of growth‐regulatory gene networks that can also be induced experimentally by treating females with testosterone (Cox et al. 2017b).

### TESTOSTERONE STRUCTURES BETWEEN‐SEX GENETIC COVARIANCE

**Figure 3:**
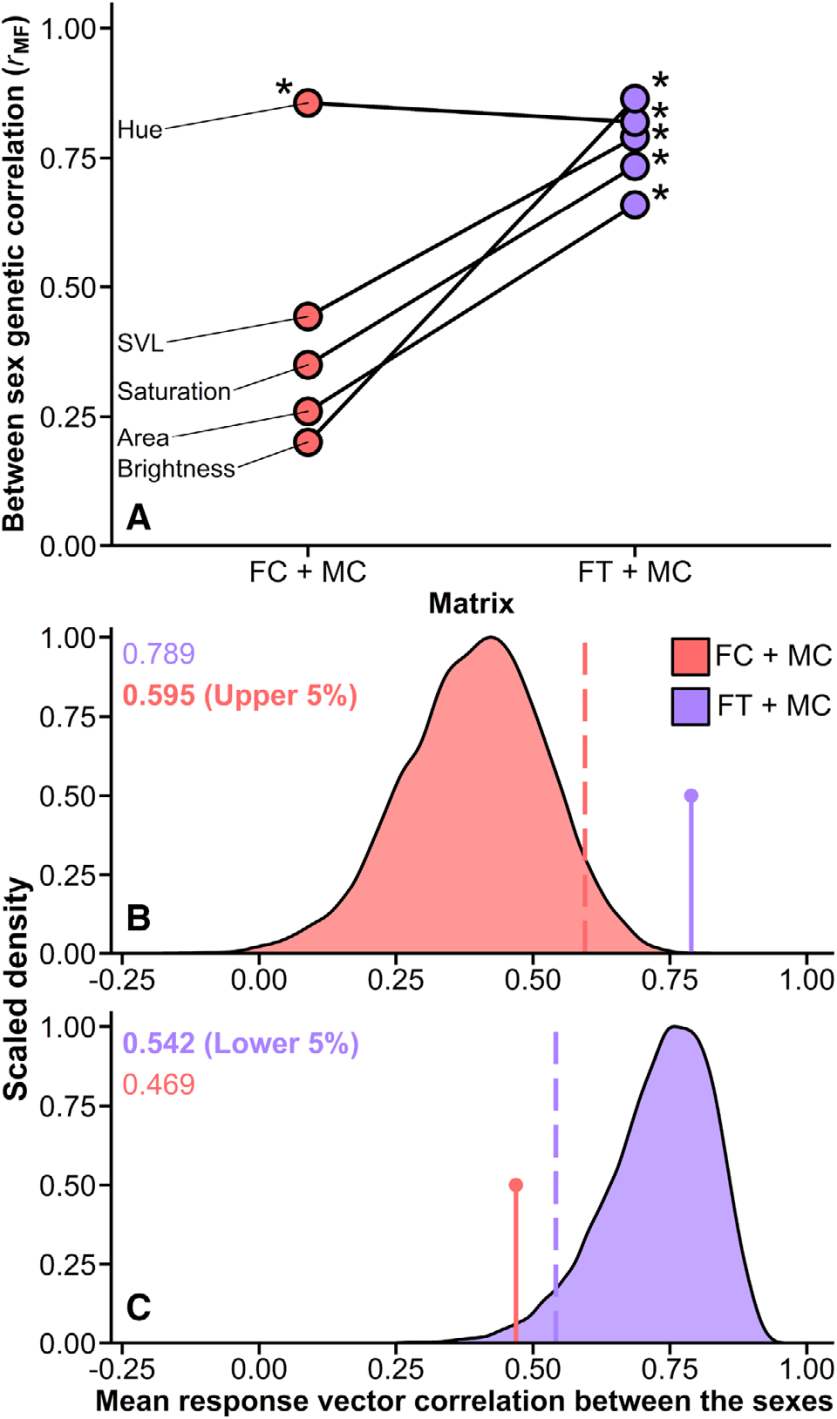
(A) Point estimates of *r*_MF_ between five homologous traits for control females and control males (FC + MC), connected to the same *r*_MF_ values for testosterone females and control males (FT + MC). Asterisks indicate estimates significantly greater than zero. (B) Distribution of 10,000 mean vector correlations between female and male responses to sexually antagonistic skewers based on the simulated distribution of the full **G** matrix (including **B**) for control females and control males. The upper 5% bound of this null distribution is shown with a dashed line. The mean vector correlation between female and male responses using the best estimate of the full **G** matrix (including **B**) for testosterone females and control males is shown with a pin and falls above the upper 5% bound. (C) The reciprocal comparison to that shown in panel B, with the mean vector correlation for control females and control males falling below the lower 5% bound of the simulated distribution for testosterone females and control males.

The inclusion of **B** significantly improved estimation of **G** for testosterone females in combination with either control males or testosterone males, but not for the combination of control females and control males (Table S12), suggesting that the elevation of testosterone in females restores underlying between‐sex genetic covariance that is naturally reduced. Only one estimate of *r*_MF_ for homologous traits was statistically greater than zero when estimated between control females and males (dewlap hue; Fig. 3A; Table S13), consistent with previous estimates of *r*_MF_ for adult anoles (Cox et al. 2017a,2017b). By contrast, all estimates of *r*_MF_ were significantly greater than zero when estimated between testosterone females and control males (Fig. 3A; Table S13), and four of five were significant between testosterone females and testosterone males (Fig. S4A; Table S13‐S14). The mean strength of *r*_MF_ was significantly lower between control females and control males than between testosterone females and control males (paired *t* = 3.13, *df* = 4, one‐tailed *P* = 0.018; Fig. 3A) or between testosterone females and testosterone males (paired *t* = 3.20, *df* = 4, one‐tailed *P* = 0.016; Fig. S4A). The mean difference in *r*_MF_ was also significantly greater than zero when comparing matrices from the simulated REML‐MVN distribution for control females and control males to matrices from either of the distributions involving testosterone females (Table S15).

High values of *r*_MF_ are thought to be the primary impediment to the evolution of sexual dimorphism (Lande 1980; Poissant et al. 2010). Our results support the prediction that *r*_MF_ can be reduced by the divergent hormonal environments in which genes are expressed in females versus males (Cox et al. 2016; Cox 2020). To our knowledge, this is the first direct experimental demonstration of an idea that traces back to Fisher (1958), but has only recently been incorporated into theory on the evolution of *r*_MF_ and its implications for intralocus sexual conflict (Badyaev 2002; Poissant and Coltman 2009; Cox et al. 2017b). Corroborating lines of evidence include the tendency for *r*_MF_ to decrease as ontogeny progresses (Poissant and Coltman 2009; Cox et al. 2017b), and the pleiotropic effects of testosterone on organismal phenotypes and underlying patterns of gene expression (Peterson et al. 2014; Mank 2017; Cox 2020).

The mean vector correlation between male and female responses to sexually antagonistic skewers was low for the natural **G** (including **B**) matrix estimated for control females and control males (*r* = 0.47), and substantially higher when estimated between testosterone females and control males (*r* = 0.79; Fig. 3B‐C; Fig. S5). Each of these values falls outside of the simulated distribution for the other matrix (Fig. 3B‐C; Table S16), and the responses of testosterone females and testosterone males to sexually antagonistic selection were also more strongly correlated than those of control males and control females (Fig. S4; Table S16), indicating that the addition of testosterone to females produced a full **G** matrix that is significantly less permissive of sex‐specific evolution under simulated sexually antagonistic selection, relative to the full **G** matrix in control animals. This agrees with a previous conclusion that the natural **B** matrix for dewlap traits is unlikely to impose a strong constraint on the short‐term evolution of sexual dimorphism (Cox et al. 2017a), and extends this conclusion by implying that natural sex differences in testosterone levels directly facilitate this weakening of between‐sex genetic constraint.

### SYNTHESIS AND IMPLICATIONS

Hormonal pleiotropy is well‐documented in this system and many others, but our study is the first to show that it structures the underlying patterns of genetic variance and covariance that shape how populations evolve in response to selection. Although this phenomenon is presumably ubiquitous, it has been largely neglected by endocrinologists and evolutionary biologists alike (Poissant and Coltman 2009; Cox et al. 2016; Cox 2020). Testosterone has often been implicated as an agent of phenotypic integration (McGlothlin and Ketterson 2008; Ketterson et al. 2009; Cox et al. 2016). We extend this framework by showing that testosterone specifically alters the additive genetic components of phenotypic variance and covariance. This implies that the experimental elevation of testosterone (which has no genetic component, unlike natural variation in testosterone levels; see Cox et al. 2016) influences phenotypic expression in ways that are dependent upon underlying genetic differences among individuals. Such differences could reflect genetic variation in (1) binding proteins that mediate the availability of free testosterone, (2) cell‐ or tissue‐specific expression of androgen receptors and cofactors necessary for initiation of transcription, (3) nucleotide motifs for androgen response elements and associated regulatory regions of androgen‐responsive target genes, and (4) coding and regulatory regions of other genes and networks underlying focal phenotypes that are located downstream of genes directly responsive to testosterone (see Cox 2020 for a review). Our results imply that the extent to which these various aspects of genetic variance and covariance are available for selection will often depend upon the endocrine backgrounds in which they occur.

The internal hormonal milieu of an individual comprises the physiological environment in which its genome is translated into phenotypes. As such, our individual‐level comparison of testosterone and control groups is conceptually similar to population‐level comparisons of **G** between different environments. Two synthetic conclusions from such studies are that environmentally induced differences in **G** are often as pronounced as those accumulated over thousands of generations of evolutionary divergence, and that evolutionary responses to selection will often differ dramatically across environments (Wood and Brodie 2015). Similarly, the “hormonal environment” in which a genome is translated into phenotypes should, by virtue of its effects on **G**, influence short‐term evolutionary trajectories. We may often overlook this feature because the hormonal environment is both highly plastic and a property of the individual, whereas **G** is a property of the population. While this is true, there are important instances in which hormonal environments differ predictably and dramatically, either at the population level or within subsets of a population. Testosterone provides a canonical example, varying with factors such as sex, age, and season.

We have shown that distinct patterns of **G** in females and males are partly due to sex differences in circulating testosterone. Likewise, although males and females share an autosomal genome, sex differences in testosterone levels can break down between‐sex genetic covariance and thereby facilitate separate evolutionary responses to sexually antagonistic selection. This evolutionary breakdown does not require upstream genetic change in testosterone production or androgen receptor expression, although such changes could contribute. It simply requires that shared autosomal genes that harbor genetic variance for phenotypes under sexually antagonistic selection become directly (e.g., *cis* regulation by androgen response elements) or indirectly (e.g., *trans* regulation by upstream genes that are responsive to testosterone) coupled to a hormone that is already sexually dimorphic. Therefore, a final key implication of our study is that a single signaling molecule, such as testosterone, provides a pleiotropic regulatory mechanism that can potentially help to alleviate a variety of evolutionary conflicts (e.g., intersexual, ontogenetic) arising from the fundamental constraint of a shared genome that experiences conflicting selection pressures between sexes or across ontogeny.

## AUTHOR CONTRIBUTIONS

R.M.C. and J.W.M. conceived the study, T.N.W. and R.M.C. designed and implemented the experiment, T.N.W. and C.D.R. collected and analyzed the data, T.N.W. and R.M.C. drafted the initial version of the manuscript and all authors contributed to later versions of the manuscript. 

## DATA ARCHIVING

Code is available on GitHub: https://github.com/ty‐wittman/evo_qg_analysis_r_code

Data are available on Dryad: https://doi.org/10.5061/dryad.1rn8pk0tf

Data are also available on Open Science Framework: https://osf.io/anbg6/

## Supporting information

Table S1. Summary of sample sizes for estimation of **P**, **G** and **B**. An initial sample of 60 sires and 120 dams were paired in a paternal half‐sibling design.
Table S2. Phenotypic effects of sex (male, female) and treatment (control, testosterone) at two time points.
Table S3. Phenotypic effects of sex (female, male), analysed separately for each treatment.
Table S4. Phenotypic effects of hormone treatment (control, testosterone), analysed separately for each sex.
Table S5. Summary of model comparisons testing for significant additive genetic variance (*V*_A_) and covariance (*Cov*_A_) in each of the four experimental groups.
Table S6. Variance‐standardized **G** matrices for each experimental group.
Table S7. Unstandardized **G** matrices for each experimental group.
Table S8. Genetic correlation matrices for each experimental group.
Table S9. Phenotypic variance‐covariance (**P**) and correlation matrices for each experimental group.
Table S10. Summary of tests for differences in **G** and genetic correlation matrices across four experimental groups.
Table S11. Summary of tests for differences in **P** and phenotypic correlation matrices across four experimental groups.
Table S12. Summary of model comparisons testing for significant additive between‐sex genetic covariance in three estimates of the full **G** matrix (including **B**) for different combinations of female and male treatment groups.
Table S13. Full variance‐standardized genetic variance‐covariance matrices (**G**), including the between‐sex covariance matrix (**B**), for three combinations of male and female treatments.
Table S14. Full unstandardized genetic variance‐covariance matrices (**G**), including the between‐sex covariance matrix (**B**), for three combinations of male and female treatments.
Table S15. Summary of tests for effects of testosterone on the magnitude of between‐sex genetic correlations (*r*_MF_) for five homologous traits (diagonals in the between‐sex genetic correlation matrices in Tables S13‐S14).
Table S16. Summary of sexually antagonistic skewers comparison of the full **G** matrix (including **B**) for control females and control males versus those estimated for testosterone females and control males or for testosterone females and testosterone males.
Figure S1. Representative images of individuals from each sex and treatment at 8 months of age (5 months post‐treatment), illustrating effects of testosterone on dewlap phenotypes.
Figure S2. Separation of experimental groups based on the first two principal components, which explain 71.5% of the variance in 5 phenotypes.
Figure S3. Comparisons of within‐sex **P** matrices across four experimental groups based on response vectors from random skewers.
Figure S4. Point estimates of *r*_MF_ between five homologous traits for control females and control males (FC + MC), connected to the same *r*_MF_ values for testosterone females and testosterone males (FT + MT).
Figure S5. Distribution of between‐sex selection vector correlations for 10,000 randomly drawn sexually antagonistic skewers (gray distribution), shown alongside corresponding distributions of 10,000 between‐sex response vector correlations derived from passing these sexually antagonist skewers through the best estimate of the **G** matrix (including **B**) derived from control females and control males (coral distribution) or from testosterone females and control males (purple distribution).
