# Supplementary material for: Hormonal pleiotropy structures genetic covariance
Source: Evol Lett. 2021 Jun 13;5(4):397–407. doi: 10.1002/evl3.240 (PMC8327939; doi:10.1002/evl3.240)
Supplement: Supplementary file 1 — Table S1. Summary of sample sizes for estimation of P, G and B. An initial sample of 60 sires and 120 dams were paired in a paternal half‐sibling design. Table S2. Phenotypic effects of sex (male, female) and treatment (control, testosterone) at two time points. Table S3. Phenotypic effects of sex (female, male), analysed separately for each treatment. Table S4. Phenotypic effects of hormone treatment (control, testosterone), analysed separately for each sex. Table S5. Summary of model comparisons testing for significant additive genetic variance (V A) and covariance (Cov A) in each of the four experimental groups. Table S6. Variance‐standardized G matrices for each experimental group. Table S7. Unstandardized G matrices for each experimental group. Table S8. Genetic correlation matrices for each experimental group. Table S9. Phenotypic variance‐covariance (P) and correlation matrices for each experimental group. Table S10. Summary of tests for differences in G and genetic correlation matrices across four experimental groups. Table S11. Summary of tests for differences in P and phenotypic correlation matrices across four experimental groups. Table S12. Summary of model comparisons testing for significant additive between‐sex genetic covariance in three estimates of the full G matrix (including B) for different combinations of female and male treatment groups. Table S13. Full variance‐standardized genetic variance‐covariance matrices (G), including the between‐sex covariance matrix (B), for three combinations of male and female treatments. Table S14. Full unstandardized genetic variance‐covariance matrices (G), including the between‐sex covariance matrix (B), for three combinations of male and female treatments. Table S15. Summary of tests for effects of testosterone on the magnitude of between‐sex genetic correlations (r MF) for five homologous traits (diagonals in the between‐sex genetic correlation matrices in Tables S13‐S14). Table S16. Summary of sexually antago [file EVL3-5-397-s001.docx]

**Supplemental Material**

**Hormonal pleiotropy structures genetic covariance**

Tyler N. Wittman, Christopher D. Robinson, Joel W. McGlothlin, and Robert M. Cox

THE MULTIVARIATE BREEDER’S EQUATION

Interest in **G** stems largely from its use in the multivariate breeder’s equation (Lande 1979; Lande and Arnold 1983):

$$\boldsymbol{\Delta}\bar{\boldsymbol{z}}=\mathbf{G}\mathbf{P}^{\boldsymbol{-1}}\mathbf{S} \mathrm{or} \left[ \begin{matrix} \Delta\bar{z}_{1} \\ \Delta\bar{z}_{2} \\ \Delta\bar{z}_{3} \end{matrix} \right]\mathbf{=}\left[ \begin{matrix} G_{11} & G_{12} & G_{13} \\ G_{12} & G_{22} & G_{23} \\ G_{13} & G_{23} & G_{33} \end{matrix} \right] \left[ \begin{matrix} P_{11} & P_{12} & P_{13} \\ P_{12} & P_{22} & P_{23} \\ P_{13} & P_{23} & P_{33} \end{matrix} \right]^{\boldsymbol{-1}}\left[ \begin{matrix} S_{1} \\ S_{2} \\ S_{3} \end{matrix} \right] (Eqn.1)$$

Here, $\boldsymbol{\Delta}\bar{\boldsymbol{z}}$ is a vector describing the between-generation evolutionary change in the means of three traits ($z_{1}, z_{2}{, z}_{3}$) in response to a vector,$\mathbf{S}$**_,_** of selection differentials ($S_{1}, S_{2}{, S}_{3}$) describing the within-generation change in trait means.$S$ includes direct selection on a trait and indirect selection on other phenotypically correlated traits. The evolutionary response therefore depends on the **P** matrix containing phenotypic variances for each trait ($P_{11}, P_{22}{, P}_{33}$) and covariances between traits ($P_{12}, P_{13}{, P}_{23}$), and on the **G** matrix containing their underlying additive genetic variances ($G_{11}{,G_{22},G}_{33}$) and covariances ($G_{12}{,G_{13},G}_{23}$). When selection is expressed using multivariate selection gradients ($\beta_{1}, \beta_{2}{, \beta}_{3}$) that account for the covariance structure in **P** to reflect only the direct component of selection, the vector $\boldsymbol{\beta}$can be substituted for $\mathbf{P}^{\boldsymbol{-1}}\mathbf{S}$:

$$\boldsymbol{\Delta}\bar{\boldsymbol{z}}=\boldsymbol{G\beta} \mathrm{or} \left[ \begin{matrix} \Delta\bar{z}_{1} \\ \Delta\bar{z}_{2} \\ \Delta\bar{z}_{3} \end{matrix} \right]\mathbf{=}\left[ \begin{matrix} G_{11} & G_{12} & G_{13} \\ G_{12} & G_{22} & G_{23} \\ G_{13} & G_{23} & G_{33} \end{matrix} \right] \left[ \begin{matrix} \beta_{1} \\ \beta_{2} \\ \beta_{3} \end{matrix} \right] (Eqn. 2)$$

To facilitate the study of sexual dimorphism, Lande (1980) modified the multivariate breeder’s equation to separate selection, genetic architecture, and evolutionary response by sex:

$\left[ \begin{matrix} \boldsymbol{\Delta}{\bar{\boldsymbol{z}}}_{\mathbf{M}} \\ \boldsymbol{\Delta}{\bar{\boldsymbol{z}}}_{\mathbf{F}} \end{matrix} \right]=\frac{1}{2}\left[ \begin{matrix} \mathbf{G}_{\mathbf{M}} & \mathbf{B} \\ \mathbf{B}^{\mathbf{T}} & \mathbf{G}_{\mathbf{F}} \end{matrix} \right] \left[ \begin{matrix} \boldsymbol{\beta}_{\mathbf{M}} \\ \boldsymbol{\beta}_{\mathbf{F}} \end{matrix} \right]\mathrm{or}\left[ \begin{matrix} \begin{matrix} \Delta\bar{z}_{1M} \\ \Delta\bar{z}_{2M} \\ \begin{matrix} \Delta\bar{z}_{3M} \\ \Delta\bar{z}_{1F} \end{matrix} \end{matrix} \\ \Delta\bar{z}_{2F} \\ \Delta\bar{z}_{3F} \end{matrix} \right]\mathbf{=}\frac{1}{2} \left[ \begin{matrix} \begin{matrix} \begin{matrix} G_{11} \\ G_{12} \end{matrix} & \begin{matrix} G_{12} \\ G_{22} \end{matrix} & \begin{matrix} G_{13} \\ G_{23} \end{matrix} \\ \begin{matrix} G_{13} \\ B_{11} \end{matrix} & \begin{matrix} G_{23} \\ B_{21} \end{matrix} & \begin{matrix} G_{33} \\ B_{31} \end{matrix} \\ \begin{matrix} B_{12} \\ B_{13} \end{matrix} & \begin{matrix} B_{22} \\ B_{23} \end{matrix} & \begin{matrix} B_{32} \\ B_{33} \end{matrix} \end{matrix} & \begin{matrix} \begin{matrix} B_{11} \\ B_{21} \end{matrix} & \begin{matrix} B_{12} \\ B_{22} \end{matrix} & \begin{matrix} B_{13} \\ B_{23} \end{matrix} \\ \begin{matrix} B_{31} \\ G_{11} \end{matrix} & \begin{matrix} B_{32} \\ G_{12} \end{matrix} & \begin{matrix} B_{33} \\ G_{13} \end{matrix} \\ \begin{matrix} G_{12} \\ G_{13} \end{matrix} & \begin{matrix} G_{22} \\ G_{23} \end{matrix} & \begin{matrix} G_{23} \\ G_{33} \end{matrix} \end{matrix} \end{matrix} \right] \left[ \begin{matrix} \beta_{1M} \\ \begin{matrix} \beta_{2M} \\ \beta_{3M} \\ \beta_{1F} \end{matrix} \\ \begin{matrix} \beta_{2F} \\ \beta_{3F} \end{matrix} \end{matrix} \right]\boldsymbol{(}Eqn. 3)$

This example also involves three traits, but $\boldsymbol{\Delta}\bar{\boldsymbol{z}}$ and $\boldsymbol{\beta}$ have been separated by sex and **G** has been expanded to include two sub-matrices describing genetic variances and covariances within each sex (**G_M_, G_F_**) as well as the between-sex matrix **B** and its transpose **B^T^**. The diagonal elements in **B** are between-sex genetic covariances for single traits ($B_{11}{,B_{22},B}_{33}$), whereas the off-diagonal elements are between-sex genetic covariances for different traits ($B_{12}{,B_{13},B}_{23}, B_{21}{,B_{31},B}_{32}$). Whereas the covariances above and below the diagonal in **G** are identical, **B** is not necessarily symmetrical in the same way because the covariance between *z*_1_ in males and *z*_2_ in females ($B_{12}$) is not necessarily the same as that between *z*_1_ in females and *z*_2_ in males ($B_{21}$).

The random skewers and sexually antagonistic skewers analyses that we use to test for matrix similarity (see main text) are based on the equations above. Comparison of **G** using random skewers is achieved by randomly drawing selection gradients to produce 10,000 random vectors representing $\boldsymbol{\beta}$, multiplying each of these 10,000 “random skewers” by an estimated or simulated **G** matrix, recording the resulting 10,000 evolutionary response vectors $\boldsymbol{\Delta}\bar{\boldsymbol{z}}$, then estimating the mean vector correlation between response vectors of the two **G** matrices being compared. Sexually antagonistic skewers are created in similar fashion, with the constraint that selection on each individual trait is constrained to be opposite in sign between the sexes (i.e., $\beta_{1F}$ is opposite in sign from $\beta_{1M}$). For analysis, 10,000 sexually antagonistic skewers are passed through the full **G** matrix (including **B** and **B^T^**) and evolutionary responses are recorded separately for $\boldsymbol{\Delta}{\bar{\boldsymbol{z}}}_{\mathbf{F}}$ and $\boldsymbol{\Delta}{\bar{\boldsymbol{z}}}_{\mathbf{M}}$, such that the mean vector correlation between male and female response vectors can be calculated. For any given **G** matrix, this mean vector correlation describes the extent to which males and females are constrained to exhibit a similar evolutionary response to selection that inherently favors sexual dimorphism in each trait. When two **G** matrices are compared, the matrix with the larger positive value of this mean vector correlation is inferred to impose relatively greater constraint on the evolution of sexual dimorphism.

ANIMAL HUSBANDRY AND BREEDING

We housed adults individually in plastic cages (30 cm x 20 cm x 20 cm; Lee’s Kritter Keeper, San Marcos, CA, USA) with a PVC pipe for perching and hiding, a strip of fiberglass mesh for basking, and a piece of outdoor carpet as substrate. We placed each cage beneath two ReptiSun 10.0 UVB bulbs (ZooMed, San Luis Obispo, CA, USA) and maintained animals on a 13L:11D photoperiod with constant 65% relative humidity and ambient temperature of 29°C during the day and 25°C at night. We misted each cage daily with deionized water. Three times per week, we fed each adult 3-5 crickets (*Gryllodes sigillatus*, 1/2” size for adult males, 3/8” size for adult females, Ghann’s Cricket Farm, Augusta, GA, USA). We dusted the crickets twice weekly with a calcium supplement (Fluker’s Repta Calcium with D_3_, Fluker Farms, Port Allen, LA, USA), and once weekly with a vitamin supplement (Fluker’s Reptile Vitamin).

For breeding, we placed one male and one female together in larger cages (40 cm x 23 cm x 32 cm; Lee’s Kritter Keeper, San Marcos, CA, USA) that contained two screen hammocks and perches, as well as a small cup of potting soil in which females could oviposit. After two weeks, we separated sires and dams and placed the cup of soil in the female’s cage. After another two weeks, we repeated this breeding protocol with the same sires and new dams to generate paternal half-sibling families. We checked each female’s cup of soil for new eggs once weekly. Anoles lay one egg at a time and typically produce an egg every 7-10 days in captivity. We assigned a unique ID to each new egg, recorded its mass, then placed it into an individual plastic container filled with moist vermiculate (1g:1g ratio vermiculite to distilled water) and covered with a transparent, perforated lid to maintain moisture while permitting gas exchange. We incubated containers at constant 28°C and 80% relative humidity with a 12L:12D light cycle in a Percival Intellus 136VL. We checked each container daily for new hatchlings, which were immediately sexed and measured for mass and snout-vent length, then housed individually in small cages identical to those described for adults (above). We fed each hatchling 3-5 crickets of 1/8” size 3 times per week until they were 3 months old, after which we fed them 3-5 crickets of 3/8” size 3 times per week, with no difference in diet between sexes. New animals hatched over a 10-month period between August 2017 and June 2018. At 3 months of age, each animal was assigned to a treatment group (see main text), then received either a testosterone implant or an empty implant as a control (see below). Sample sizes are presented in Table S1.

IMPLANT CONSTRUCTION AND TESTOSTERONE MANIPULATION

We made implants by dissolving testosterone (T-1500, Sigma-Aldrich Inc., St. Louis. MO, USA) in dimethyl sulfoxide (DMSO) at a concentration of 100 µg testosterone per µL DMSO, then injecting 1 µl of this solution into a 4-mm section of Silastic tubing (Dow Corning, Midland, MI, USA: 1.47 mm I.D. x 1.96 mm O.D.) which we had previously sealed on one end using 100% silicone gel. After loading the hormone solution, we sealed the open end of the implant with silicone gel, then waited 48-72 h for the silicone to cure and for the DMSO to diffuse out of the implant, leaving 100 µg of crystalized testosterone within the lumen of the sealed Silastic implant. As an experimental control, we produced empty implants by following the same procedure with 1 µl pure DMSO in place of the testosterone solution. This hormone dose and implant size were selected because they have been shown to increase circulating testosterone in juvenile anoles of comparable size while maintaining plasma levels within the natural physiological range for adult males (Cox et al. 2015).

We fasted lizards for 24 h prior to surgery, then gave each animal a 2-µl intra-peritoneal injection of bupivacaine (2.5 mg mL^-1^) at the site of incision as an anesthetic and analgesic. We then cooled lizards at -20°C for 4-5 min and immobilized them atop a partially thawed chemical ice pack. We sanitized the incision site with alternating wipes of 70% isopropyl alcohol and 4% chlorhexidine gluconate solution. Surgical instruments were heat-sterilized in a Germinator 500 bead sterilizer (DS-501, Roboz Surgical Instrument Co., Gaithersbur, MD, USA) before each surgery. We made a 3-mm incision in the ventral abdomen and inserted an ethanol-sterilized implant (testosterone or empty control) into the coelomic cavity and closed the incision with cyanoacrylate adhesive (VetClose®, Butler Schein Animal Health, Dublin, OH, USA).

MEASURMENT OF DEWLAP PHENOTYPES

We manually extended the dewlap by pulling out the second ceratobranchial cartilage with forceps (Fig. S1), then took a digital photograph (Canon EOS Rebel T3i with 100-mm macro lens) from a set distance against a standardized background (graph paper, 5-mm grids) under standardized lighting (FE30050-10 28W fluorescent photography bulb in reflecting hood at set distance and angle) next to a color standard (Kodak Gray Scale and Color Control patches). We measured dewlap traits using the Fiji distribution (Schindelin et al. 2012) of ImageJ (Schneider et al. 2012). We uploaded images into ImageJ and set the scale of measurement using the 5-mm grids of the graph paper. We measured area (mm^2^) by outlining the dewlap from its anterior projection from chin to its posterior attachment to the venter using the “polygon” tool. To quantify the color of the dewlap, we used the “oval” tool to define a circle in the center of the dewlap, with diameter of the circle equal to 1/3 the width of the dewlap, providing a consistent measure of the “center” of each dewlap despite variation in its absolute size. We used the “color histogram” function to extract the mean red, green, and blue values for the selected area, then transformed these values into hue (primary color reflected, measured on a 360° color wheel), saturation (purity of the hue, 0% = achromatic, 100% = pure color), and brightness (relative to maximum possible for color of the same hue and saturation, 0% = black, 100% = white-tint-pure color) using the rgb2hsv function of the package gDevices within R3.6.2 (R Core Team 2019).

PRINCIPAL COMPONENT ANALYSIS OF PHENOTYPES

Univariate tests for effects of sex and age on each individual phenotype are presented in Tables S2-S4. To holistically assess sex and treatment effects on phenotypes at 8 months of age (5 months post-treatment), we used PCA to visualize multivariate treatment differences in reduced phenotypic space (Fig. S2). The first principal component (PC1) explained 49% of phenotypic variance, PC2 explained 22.5%, PC3 explained 17.3%, PC4 explained 8.2%, and PC5 explained the remaining 3% of phenotypic variance. PC1 was defined primarily by positive loadings for size variables (ln SVL and ln dewlap area) and negative loadings for dewlap brightness (Fig. S2). PC2 was defined primarily by positive loadings for dewlap saturation and negative loadings for dewlap hue (Fig. S2). We used ANOVA to test the extent to which main effects of sex, treatment, and their interaction explained individual variation along PC1 and PC2. For PC1, there was a significant effect of sex (*F*_1,934_ = 3499.3, *P* < 0.001), treatment (*F*_1,934_ = 295.8, *P* < 0.001), and their interaction (*F*_1,934_ = 784.4, *P* < 0.001). Males had higher PC1 scores than females (i.e., males were larger and had larger and less bright dewlaps than females) and testosterone strongly increased PC1 scores in females (i.e., testosterone increased SVL and dewlap area while decreasing dewlap brightness, Fig. S2). For PC2, there was no effect of sex (*F*_1,934_ = 1.94, *P* = 0.164), but there was a significant treatment effect (*F*_1,934_ = 114.2, *P* < 0.001) and a weak interaction between sex and treatment (*F*_1,934_ = 6.537, *P* = 0.011). Testosterone increased PC2 scores, meaning that testosterone increased dewlap saturation and decreased dewlap hue relative to control animals (Fig. S2).

ANIMAL MODEL AND ESTIMATION OF **G**

We used linear mixed effect models (i.e., animal models) to estimate additive genetic variances and covariances. The animal model incorporates complex pedigree structures, fits both fixed and additional random effects (e.g., hatch month in our analyses), and provides estimates of additive genetic variance and covariance that are unbiased for unbalanced data (Kruuk 2004). The general formula for the univariate animal model as applied in our study is:

$$\mathbf{y}=\mathbf{Xb}+\mathbf{Z}_{\mathbf{1}}\mathbf{a}+\mathbf{Z}_{\mathbf{2}}\mathbf{h}+\mathbf{e} (Eqn. 4)$$

Here, $\mathbf{y}$ is a vector of individual trait values with a length equal to the number of individuals, $\mathbf{X}$ is a design matrix linking individual observations to fixed effects, $\mathbf{b}$ is a vector of fixed effects (in our case, this only includes the population mean trait value), $\mathbf{Z}_{\mathbf{1}}$ is a design matrix linking individual observations to the random additive genetic effect (all individuals with observations are included in the pedigree, so $\mathbf{Z}_{\mathbf{1}}$ is an identity matrix), $\mathbf{a}$ is a vector of random additive genetic effects (composed of additive genetic variance and the numerator relationship matrix), $\mathbf{Z}_{\mathbf{2}}$ is a design matrix linking individual observations to the random effect of hatch month, $\mathbf{h}$ is a vector containing the random effect of hatch month, and $\mathbf{e}$ is the vector of residual error. For multivariate estimates of **G** within each sex or treatment, we extended eqn. 4 to include five traits. For estimates of the full **G**, including **B**, we modeled homologous traits in males and females as separate traits (as in eqn. 3), creating a 10-trait matrix (Wolak et al. 2015).

LIKELIHOOD RATIO TESTS FOR SIGNIFICANT GENETIC (CO)VARIANCE

To test for significant additive genetic variance and covariance, we used likelihood ratio tests to compare our estimates of the full **G** matrix for each experimental group with simpler models setting covariances to zero or excluding additive genetic effects entirely (Table S5). The test statistic was calculated as twice the difference in log likelihood between models and tested against a chi-squared distribution. For each experimental group, the full **G** was preferred over simpler models, confirming the presence of significant additive genetic variance and covariance (Table S5). We used a similar approach to test for significant between-sex genetic covariance for combinations of male and female groups by comparing estimates of the full **G** (including **B**) with simpler models setting the elements in **B** to zero (Table S12). In the matrix estimated for control females and control males, the inclusion of **B** did not significantly improve the model, indicating weak between-sex genetic covariance in this “natural” condition. However, models including **B** were preferred over simpler models without **B** in matrices estimated for testosterone females and either control males or testosterone males (Table S12), indicating that between-sex genetic covariance is only significant when both sexes have elevated testosterone.

COMPARISON OF PHENOTYPIC COVARIANCE MATRICES (**P**)

The comparisons presented in the main text focus on **G** and **B**, which contain additive genetic variances and covariances. Hormonal pleiotropy is also predicted to structure **P**, the matrix of phenotypic variances and covariances (McGlothlin and Ketterson 2008; Ketterson et al. 2009; Cox et al. 2016; Cox 2020), potentially by altering non-additive or non-genetic components of phenotypic variance and covariance that are not captured by **G**. Therefore, we also used WOMBAT (Meyer 2007) to estimate **P** for each experimental group, then compared each pair of **P** matrices using random skewers as described for comparison of **G** (see main text), with the caveat that vectors derived from **P** do not correspond to “evolutionary response” per se. Because **P** is estimated with less error than **G**, null distributions for each matrix are relatively narrow, such that **P** was statistically distinct between all groups except control and testosterone males and testosterone females and testosterone males (Fig. S3). Mean vector correlations were quantitatively higher between testosterone females and either male group (0.97 < *r* < 0.98) than between control females and either male group (0.88 < *r* < 0.92; Fig. S3; Table S10, section A).

COMPARISON OF UNSTANDARDIZED AND VARIANCE-STANDARDIZED **G** MATRICES

The comparisons presented in the main text were conducted after variance-standardizing **G** by dividing each element in the matrix by its corresponding phenotypic variance (Table S6). This method prevents differences in the units in which traits are measured from predisposing some traits to contribute disproportionately to the size of **G** and the associated evolutionary response. The variance-standardized **G** can be used in the multivariate breeder’s equation (Eqns. 2-3) when the selection gradients forming the vector $\boldsymbol{\beta}$ are estimated using traits that have been re-scaled to mean of zero and unit variance. In this formulation, the response to selection, $\boldsymbol{\Delta}\bar{\boldsymbol{z}}$, is in units of phenotypic standard deviations. As a complementary approach, we repeated all of the same matrix comparisons using unstandardized estimates of **G** (Table S7). These two approaches are compared in Tables S10 and S16. Results are qualitatively similar using either approach.

COMPARISON OF CORRELATION MATRICES

The analyses presented above and in the main text focus on matrices whose elements are phenotypic or genetic variances (on the diagonal) or covariances (off the diagonal), as illustrated in Eqns. 1-3. We also estimated phenotypic and genetic correlation matrices, which replace covariances with correlations (covariances divided by geometric mean variances). To compare phenotypic (Table S9) and genetic (Table S8) correlation matrices estimated within each group, we excluded diagonal elements in **P** and **G** and analyzed matrices comprised of the 10 unique correlations between 5 traits. We used two methods to compare correlation matrices.

First, we calculated the Mantel correlation between each pair of matrices (the Pearson product-moment correlation between the 10 unique correlations in each matrix) using R 3.5.3 code available on github (see Data Accessibility, main manuscript). We compared these Mantel correlations to null distributions of Mantel correlations between the best estimate of each correlation matrix and each of the 10,000 simulated matrices in the REML-MVN distribution for that experimental group, after first transforming these simulated **P** or **G** matrices into correlation matrices using the cov2cor function in R 3.5.3 (R Core Team 2019). Our method is similar to that of Goodnight and Schwartz (1997) but uses the REML-MVN method instead of their bootstrapping method to derive a null distribution. We tested whether the Mantel correlations between the best estimates of any two matrices fell below the lower 5% bound of the null distribution for either matrix. Mantel comparisons between genetic correlation matrices are reported in Table S10, those for phenotypic correlation matrices are in Table S11.

Second, we used a modified version of the T method (Roff et al. 2012) to ask whether the mean absolute value of the difference between individual elements in two matrices was greater than expected from sampling error. As with the Mantel method described above, we created a null distribution for each experimental group by calculating the mean absolute value of the differences in each element of the matrix between the best estimate of a matrix and each of the 10,000 matrices in its REML-MVN sampling distribution. We then tested whether the mean absolute difference in elements between the best estimates of any two matrices fell below the lower 5% bound of the null distribution for either matrix. These “modified T method” comparisons between genetic correlation matrices are reported in Table S10, those for phenotypic correlation matrices are in Table S11.

**Supplemental References**

Cox, R.M., McGlothlin, J.W. and Bonier, F. 2016. Hormones as mediators of phenotypic and genetic integration: An evolutionary genetics approach. *Integrative and Comparative Biology* **56:** 126-137.

Cox, R.M. 2020. Sex steroids as mediators of phenotypic integration, genetic correlations, and evolutionary transitions. *Molecular and Cellular Endocrinology* **502:** 110628.

Goodnight, C.J. and Schwartz, J.M. 1997. A bootstrap comparison of genetic covariance matrices. *Biometrics* **53:** 1026-1039.

Ketterson, E.D., Atwell, J.W. and McGlothlin, J.W. 2009. Phenotypic integration and independence: Hormones, performance, and response to environmental change. *Integrative and Comparative Biology* **49:** 365-379.

Kruuk, L.E.B. 2004. Estimating genetic parameters in natural populations using the ‘animal model’. *Philosophical Transactions of the Royal Society of London. Series B: Biological Sciences* **359:** 873-890.

Lande, R. 1979. Quantitative Genetic Analysis of Multivariate Evolution, Applied to Brain: Body Size Allometry. *Evolution* **33:** 402-416.

Lande, R. 1980. Sexual dimorphism, sexual selection, and adaptation in polygenic characters. *Evolution* **34:** 292-307.

Lande, R. and Arnold, S.J. 1983. The measurement of selection on correlated characters. *Evolution* **37:** 1210-1226.

McGlothlin, J.W. and Ketterson, E.D. 2008. Hormone-mediated suites as adaptations and evolutionary constraints. *Philosophical Transactions of the Royal Society B: Biological Sciences* **363:** 1611-1620.

Meyer, K. 2007. WOMBAT - A tool for mixed model analyses in quantitative genetics by restricted maximum likelihood. *Journal of Zhejiang University SCIENCE B* **8:** 815-821.

Roff, D.A., Prokkola, J.M., Krams, I. and Rantala, M.J. 2012. There is more than one way to skin a G matrix. *Journal of Evolutionary Biology* **25:** 1113-1126.

Schindelin, J., Agranda-Carreras, I., Frise, E., Kaynig, V., Longair, M., Pietzsch, T., Preilbisch, S., Rueden, C., Saalfeld, S., Schmid, B., Tinevez, J.-Y., White, D.J., Hartenstein, V., Eliceiri, K., Tomancak, P. and Cardona, A. 2012. Fiji: An open-source platform for biological-image analysis. *Nature Methods* **9:** 676-682.

Schneider, C.A., Rasbabd, W.S. and Eliceiri, K.W. 2012. NIH Image to ImageJ: 25 years of image analysis. *Nature Methods* **9:** 671-675.

Wolak, M.E., Roff, D.A. and Fairbairn, D.J. 2015. Are we underestimating genetic variances of dimorphic traits? *Ecology and Evolution* **5:** 590-597.

**Supplemental Tables**

**Table S1.** Summary of sample sizes for estimation of **P**, **G** and **B**. An initial sample of 60 sires and 120 dams were paired in a paternal half-sibling design. Because some pairs did not produce offspring, a total of 938 individual progeny were included in the final experiment, representing 57 sires and 104 dams. Values in the table report the mean, minimum, and maximum number of half siblings and full siblings within each experimental group.

|  |  |  |  |  | **Half-sib family size** | | |  | **Full-sib family size** | | |
| --- | --- | --- | --- | --- | --- | --- | --- | --- | --- | --- | --- |
| **Sex** | **Treatment** | **Individuals** | **Sires** | **Dams** | **Mean** | **Min.** | **Max.** |  | **Mean** | **Min.** | **Max.** |
|  |  |  |  |  |  |  |  |  |  |  |  |
| Female | Control | 224 | 55 | 92 | 4.00 | 1 | 10 |  | 2.43 | 1 | 8 |
|  | Testosterone | 243 | 57 | 94 | 4.26 | 1 | 10 |  | 2.58 | 1 | 6 |
| Male | Control | 233 | 52 | 89 | 4.48 | 1 | 9 |  | 2.61 | 1 | 6 |
|  | Testosterone | 238 | 56 | 96 | 4.25 | 1 | 10 |  | 2.47 | 1 | 6 |
| **Both** | **Both** | **938** | **57** | **104** | **16.46** | **2** | **34** |  | **9.01** | **1** | **22** |
|  |  |  |  |  |  |  |  |  |  |  |  |

**Table S2.** Phenotypic effects of sex (male, female) and treatment (control, testosterone) at two time points. Treatment is included in “Pre-treat (3 mo)” models to confirm the absence of phenotypic differences prior to manipulation. Interactions between sex and treatment were never significant in pre-treatment analyses and are therefore not shown in the table. Hatch month is included as a random effect to account for overall variation across the 10-month study. All models also included random effects of sire and dam (nested within sire), which are not shown here. Interactions between sex and ln SVL were tested in all models but only retained when significant. Ln SVL is not included in “post-treat (8 mo)” analyses of dewlap traits because males and females did not overlap in this size covariate.

| **Time point (age)** |  | **Sex** | |  | **Treatment** | |  | **Ln SVL** | |  | **Sex*Ln SVL** | |
| --- | --- | --- | --- | --- | --- | --- | --- | --- | --- | --- | --- | --- |
| **Phenotype** | ***N*** | ***χ ^2^*** | ***P*** |  | ***χ ^2^*** | ***P*** |  | ***χ ^2^*** | ***P*** |  | ***χ ^2^*** | ***P*** |
|  |  |  |  |  |  |  |  |  |  |  |  |  |
| Pre-treat (3 mo) |  |  |  |  |  |  |  |  |  |  |  |  |
| Ln SVL | 289 | **23.82** | **< 0.0001** |  | 0.75 | 0.3864 |  | — | — |  | — | — |
| Ln area | 289 | **222.72** | **< 0.0001** |  | 0.70 | 0.4017 |  | **144.38** | **< 0.0001** |  | **6.08** | **0.0136** |
| Hue | 289 | 0.78 | 0.3767 |  | 0.32 | 0.5718 |  | 3.80 | 0.0510 |  | — | — |
| Saturation | 289 | **50.93** | **< 0.0001** |  | 0.25 | 0.6156 |  | 1.28 | 0.2578 |  | — | — |
| Brightness | 289 | **7.53** | **0.0060** |  | 0.11 | 0.7345 |  | 1.27 | 0.2590 |  | **6.96** | **0.0080** |
|  |  |  |  |  |  |  |  |  |  |  |  |  |
|  |  | **Sex** | |  | **Treatment** | |  | **Sex*Treatment** | |  | **Sex*Ln SVL** | |
|  |  | ***χ ^2^*** | ***P*** |  | ***χ ^2^*** | ***P*** |  | ***χ ^2^*** | ***P*** |  | ***χ ^2^*** | ***P*** |
|  |  |  |  |  |  |  |  |  |  |  |  |  |
| Post-treat (8 mo) |  |  |  |  |  |  |  |  |  |  |  |  |
| Ln SVL | 938 | **2128.30** | **< 0.0001** |  | **73.11** | **< 0.0001** |  | **348.16** | **< 0.0001** |  | — | — |
| Ln area | 938 | **5599.27** | **< 0.0001** |  | **810.01** | **< 0.0001** |  | **665.82** | **< 0.0001** |  | — | — |
| Hue | 938 | **83.05** | **< 0.0001** |  | **4.71** | **0.0298** |  | **58.82** | **< 0.0001** |  | — | — |
| Saturation | 938 | **119.52** | **< 0.0001** |  | **156.51** | **< 0.0001** |  | **12.61** | **0.0004** |  | — | — |
| Brightness | 938 | **361.39** | **< 0.0001** |  | **294.69** | **< 0.0001** |  | **306.84** | **< 0.0001** |  | — | — |
|  |  |  |  |  |  |  |  |  |  |  |  |  |

**Table S3.** Phenotypic effects of sex (female, male), analysed separately for each treatment. Hatch month was included as a random effect to account for overall variation across the 10-month study. All models also include random effects of sire and dam (nested within sire), which are not shown. Sex differences were pronounced in control treatments but reduced or even eliminated when both sexes received testosterone treatments, particularly for dewlap traits (compare magnitude of χ**^2^** statistics between Control and Testosterone groups).

| **Treatment** |  | **Sex difference** | |
| --- | --- | --- | --- |
| **Phenotype** | ***N*** | ***χ ^2^*** | ***P*** |
|  |  |  |  |
| Control |  |  |  |
| Ln SVL | 457 | **2644.60** | **< 0.0001** |
| Ln area | 457 | **6016.00** | **< 0.0001** |
| Hue | 457 | **111.37** | **< 0.0001** |
| Saturation | 457 | **97.27** | **< 0.0001** |
| Brightness | 457 | **694.85** | **< 0.0001** |
|  |  |  |  |
| Testosterone |  |  |  |
| Ln SVL | 481 | **1166.60** | **< 0.0001** |
| Ln area | 481 | **1221.30** | **< 0.0001** |
| Hue | 481 | 2.98 | 0.0800 |
| Saturation | 481 | **29.97** | **< 0.0001** |
| Brightness | 481 | 2.91 | 0.0800 |
|  |  |  |  |

**Table S4.** Phenotypic effects of hormone treatment (control, testosterone), analysed separately for each sex. Hatch month is included as a random effect to account for overall variation across the 10-month study. All models also included random effects of sire and dam (nested within sire), which are not shown. Ln SVL is included in analyses of dewlap traits, such that treatment differences are standardized for treatment effects on body size. Testosterone treatment influenced all phenotypes in both sexes. Treatment effects were similar when using raw phenotypic values for SVL and dewlap area without ln transformation.

| **Sex** |  | **Treatment** | |  | **Ln SVL** | |  | **Treatment*SVL** | |
| --- | --- | --- | --- | --- | --- | --- | --- | --- | --- |
| **Phenotype** | ***N*** | ***χ ^2^*** | ***P*** |  | ***χ ^2^*** | ***P*** |  | ***χ ^2^*** | ***P*** |
|  |  |  |  |  |  |  |  |  |  |
| Female |  |  |  |  |  |  |  |  |  |
| Ln SVL | 467 | **66.04** | **< 0.0001** |  | — | — |  | — | — |
| Ln area | 467 | **1390.54** | **< 0.0001** |  | **94.25** | **< 0.0001** |  | 0.45 | 0.5007 |
| Hue | 467 | **9.68** | **0.0018** |  | 1.75 | 0.1849 |  | 0.22 | 0.6363 |
| Saturation | 467 | **122.35** | **< 0.0001** |  | 0.05 | 0.8179 |  | **4.16** | **0.0413** |
| Brightness | 467 | **403.92** | **< 0.0001** |  | **14.13** | **0.0001** |  | 0.01 | 0.984 |
|  |  |  |  |  |  |  |  |  |  |
| Male |  |  |  |  |  |  |  |  |  |
| Ln SVL | 471 | **318.90** | **< 0.0001** |  | — | — |  | — | — |
| Ln area | 471 | **94.83** | **< 0.0001** |  | **161.23** | **< 0.0001** |  | **54.12** | **< 0.0001** |
| Hue | 471 | **12.18** | **0.0004** |  | **14.17** | **< 0.0001** |  | 1.91 | 0.1666 |
| Saturation | 471 | **3.93** | **0.0470** |  | **31.74** | **< 0.0001** |  | 1.05 | 0.3040 |
| Brightness | 471 | **11.40** | **0.0007** |  | **29.41** | **< 0.0001** |  | 2.52 | 0.1100 |
|  |  |  |  |  |  |  |  |  |  |

**Table S5.** Summary of model comparisons testing for significant additive genetic variance (*V*_A_) and covariance (*Cov*_A_) in each of the four experimental groups. In each group, models that included *V*_A_ and *Cov*_A_ (i.e., the full **G** matrix) were preferred over simpler models that set *Cov*_A_ = 0 or excluded additive effects entirely, thus confirming significant additive genetic variance and covariance in each of the four estimates of **G**. Chi-squared values are calculated as twice the difference in log likelihood between models and are shown for each of the reduced models in comparison to the preferred full **G** model.

| **Experimental group** | **Matrix** | **Log Likelihood** | **df** | ***χ* ^2^** | ***P*** |
| --- | --- | --- | --- | --- | --- |
|  |  |  |  |  |  |
| Control Female | Full **G** (*V*_A_ + *Cov*_A_) | –2735.24 | — | — | — |
|  | Only *V*_A_ (*Cov*_A_ = 0) | –2768.28 | 10 | 66.08 | < 0.00001 |
|  | Null (*V*_A_ = 0, *Cov*_A_ = 0) | –3176.31 | 5 | 816.07 | < 0.00001 |
|  |  |  |  |  |  |
| Testosterone Female | Full **G** (*V*_A_ + *Cov*_A_) | –3079.18 | — | — | — |
|  | Only *V*_A_ (*Cov*_A_ = 0) | –3121.10 | 10 | 83.84 | < 0.00001 |
|  | Null (*V*_A_ = 0, *Cov*_A_ = 0) | –3283.30 | 5 | 324.39 | < 0.00001 |
|  |  |  |  |  |  |
| Control Male | Full **G** (*V*_A_ + *Cov*_A_) | –2849.98 | — | — | — |
|  | Only *V*_A_ (*Cov*_A_ = 0) | –2895.20 | 10 | 90.44 | < 0.00001 |
|  | Null (*V*_A_ = 0, *Cov*_A_ = 0) | –3061.00 | 5 | 331.60 | < 0.00001 |
|  |  |  |  |  |  |
| Testosterone Male | Full **G** (*V*_A_ + *Cov*_A_) | –2933.93 | — | — | — |
|  | Only *V*_A_ (*Cov*_A_ = 0) | –2988.72 | 10 | 109.59 | < 0.00001 |
|  | Null (*V*_A_ = 0, *Cov*_A_ = 0) | –3355.04 | 5 | 732.64 | < 0.00001 |
|  |  |  |  |  |  |

**Table S6.** Variance-standardized **G** matrices for each experimental group. Elements on the diagonal are genetic variances (from Table S7) divided by phenotypic variances (from Table S9), which corresponds to narrow-sense heritability (*h^2^*). Elements off the diagonal are genetic covariances divided by mean phenotypic variances (from Tables S7 and S9). Numbers in parentheses are 1 SEM. **Bold** font indicates estimates >2 SEM above or below zero, approximating statistical significance. Note that the magnitudes of the genetic variances (and covariances) are independent of the units in which the phenotypes are measured (compare to Table S7). For this reason, these variance-standardized **G** matrices were used for primary analyses, though unstandardized matrices yielded equivalent results (Table S10).

| Variance-standardized **G** matrix: **Control Females** | | | | | |  | Variance-standardized **G** matrix: **Control Males** | | | | |
| --- | --- | --- | --- | --- | --- | --- | --- | --- | --- | --- | --- |
|  | Ln SVL | Ln Area | Hue | Bright | Sat |  | Ln SVL | Ln Area | Hue | Bright | Sat |
| Ln SVL | 0.263  (0.136) | 0.103  (0.105) | 0.144  (0.120) | 0.021  (0.058) | 0.179  (0.110) |  | 0.155  (0.079) | 0.070  (0.059) | -0.039  (0.085) | -0.062  (0.067) | -0.007  (0.081) |
| Ln Area | 0.103  (0.105) | **0.296**  **(0.139)** | -0.043  (0.120) | -0.038  (0.061) | 0.205  (0.117) |  | 0.070  (0.059) | 0.081  (0.062) | -0.129  (0.079) | -0.031  (0.057) | 0.058  (0.073) |
| Hue | 0.144  (0.120) | -0.043  (0.120) | **0.675**  **(0.211)** | **0.276**  **(0.103)** | -0.131  (0.131) |  | -0.039  (0.085) | -0.129  (0.079) | **0.591**  **(0.184)** | 0.064  (0.097) | **-0.311**  **(0.142)** |
| Bright | 0.021  (0.058) | -0.038  (0.061) | **0.276**  **(0.103)** | **0.129**  **(0.062)** | -0.129  (0.078) |  | -0.062  (0.068) | -0.031  (0.057) | 0.064  (0.097) | **0.238**  **(0.105)** | **0.225**  **(0.095)** |
| Sat | 0.179  (0.110) | 0.205  (0.117) | -0.131  (0.131) | -0.129  (0.078) | **0.529**  **(0.177)** |  | -0.007  (0.081) | 0.058  (0.073) | **-0.311**  **(0.142)** | **0.225**  **(0.095)** | **0.477**  **(0.153)** |
|  |  |  |  |  |  |  |  |  |  |  |  |
| Variance-standardized **G** matrix: **Testosterone Females** | | | | | |  | Variance-standardized **G** matrix: **Testosterone Males** | | | | |
| Ln SVL | 0.126  (0.077) | 0.076  (0.077) | -0.049  (0.096) | -0.107  (0.071) | -0.104  (0.074) |  | 0.093  (0.077) | 0.017  (0.055) | -0.127  (0.089) | -0.070  (0.087) | 0.067  (0.068) |
| Ln Area | 0.076  (0.077) | 0.247  (0.127) | -0.145  (0.105) | **-0.242**  **(0.105)** | -0.033  (0.067) |  | 0.017  (0.055) | 0.170  (0.093) | **-0.239**  **(0.101)** | **-0.199**  **(0.097)** | -0.010  (0.075) |
| Hue | -0.049  (0.090) | -0.145  (0.105) | **0.601**  **(0.196)** | 0.191  (0.109) | -0.193  (0.101) |  | -0.127  (0.089) | **-0.239**  **(0.101)** | **0.579**  **(0.187)** | **0.354**  **(0.131)** | -0.154  (0.098) |
| Bright | -0.107  (0.071) | **-0.242**  **(0.105)** | 0.191  (0.109) | **0.311**  **(0.120)** | 0.106  (0.083) |  | -0.070  (0.087) | **-0.199**  **(0.097)** | **0.354**  **(0.131)** | **0.439**  **(0.168)** | 0.109  (0.111) |
| Sat | -0.104  (0.074) | -0.033  (0.067) | -0.193  (0.101) | 0.106  (0.083) | **0.256**  **(0.106)** |  | 0.067  (0.068) | -0.010  (0.075) | -0.154  (0.098) | 0.109  (0.111) | **0.275**  **(0.135)** |
|  |  |  |  |  |  |  |  |  |  |  |  |

**Table S7.** Unstandardized **G** matrices for each experimental group. Elements on the diagonal are genetic variances, elements off the diagonal are genetic covariances. Numbers in parentheses are 1 SEM. **Bold** font indicates estimates >2 SEM above or below zero, approximating statistical significance. Note that the magnitudes of the genetic variances (and covariances) are strongly associated with the units in which the phenotypes are measured (e.g., smaller for ln-transformed size traits, larger for color traits measured in degrees or percentages). For this reason, variance-standardized **G** matrices (Table S6) were used for primary analyses, though both methods yielded equivalent results (Table S10).

| Unstandardized **G** matrix: **Control Females** | | | | | |  | Unstandardized **G** matrix: **Control Males** | | | | |
| --- | --- | --- | --- | --- | --- | --- | --- | --- | --- | --- | --- |
|  | Ln SVL | Ln Area | Hue | Bright | Sat |  | Ln SVL | Ln Area | Hue | Bright | Sat |
| Ln SVL | 0.00040  (0.00022) | 0.00096  (0.00097) | 0.02306  (0.01919) | 0.00822  (0.02273) | 0.06306  (0.03877) |  | 0.00049  (0.00025) | 0.00154  (0.00129) | -0.00754  (0.01624) | -0.02882  (0.03162) | -0.00422  (0.04750) |
| Ln Area | 0.00096  (0.00097) | **0.01648**  **(0.00771)** | -0.04146  (0.11586) | -0.08779  (0.14155) | 0.43483  (0.24916) |  | 0.00154  (0.00129) | 0.01220  (0.00937) | -0.16970  (0.10395) | -0.09845  (0.18429) | 0.23529  (0.29539) |
| Hue | 0.02306  (0.01919) | -0.04146  (0.11586) | **11.2295**  **(3.5096)** | **11.1573**  **(4.1555)** | -4.7918  (4.8067) |  | -0.00754  (0.01624) | -0.16970  (0.10395) | **6.8299**  **(2.1303)** | 1.8069  (2.7342) | **-11.0493**  **(5.0529)** |
| Bright | 0.00822  (0.02273) | -0.08779  (0.14155) | **11.1573**  **(4.1555)** | **12.7337**  **(6.1428)** | -11.5435  (6.9874) |  | -0.02882  (0.03162) | -0.09845  (0.18429) | 1.8069  (2.7342) | **16.4964**  **(7.2745)** | **19.5657**  **(8.2483)** |
| Sat | 0.06306  (0.03877) | 0.43483  (0.24916) | -4.7918  (4.8067) | -11.5435  (6.9874) | **42.8657**  **(14.3731)** |  | -0.00422  (0.04750) | 0.23529  (0.29539) | **-11.0493**  **(5.0529)** | **19.5657**  **(8.2483)** | **52.1729**  **(19.5437)** |
|  |  |  |  |  |  |  |  |  |  |  |  |
| Unstandardized **G** matrix: **Testosterone Females** | | | | | |  | Unstandardized **G** matrix: **Testosterone Males** | | | | |
| Ln SVL | 0.00035  (0.00022) | 0.00142  (0.00144) | -0.00800  (0.01457) | -0.05469  (0.03662) | -0.06700  (0.04764) |  | 0.00036  (0.00030) | 0.00029  (0.00095) | -0.02440  (0.01718) | -0.03580  (0.04439) | 0.04006  (0.04073) |
| Ln Area | 0.00142  (0.00144) | 0.03103  (0.01607) | -0.15769  (0.11400) | **-0.83351**  **(0.36098)** | -0.14234  (0.29912) |  | 0.00029  (0.00095) | 0.01286  (0.00708) | **-0.20215**  **(0.08515)** | **-0.44859**  **(0.21851)** | -0.02613  (0.19707) |
| Hue | -0.00800  (0.01457) | -0.15769  (0.11400) | **5.6522**  **(1.8431)** | 5.6958  (3.2370) | -7.1979  (3.7601) |  | -0.02440  (0.01718) | **-0.20215**  **(0.08515)** | **5.4729**  **(1.7648)** | **8.9045**  **(3.2847)** | -4.4887  (2.8601) |
| Bright | -0.05469  (0.03662) | **-0.83351**  **(0.36098)** | 5.6958  (3.2370) | **29.3377**  **(11.3314)** | 12.4895  (9.7609) |  | -0.03580  (0.04439) | **-0.44859**  **(0.21851)** | **8.9045**  **(3.2847)** | **29.2964**  **(11.2129)** | 8.4475  (8.6339) |
| Sat | -0.06700  (0.04764) | -0.14234  (0.29912) | -7.1979  (3.7601) | 12.4895  (9.7609) | **37.8326**  **(15.6573)** |  | 0.04006  (0.04073) | -0.02613  (0.19707) | -4.4887  (2.8601) | 8.4475  (8.6339) | **24.8055**  **(11.2552)** |
|  |  |  |  |  |  |  |  |  |  |  |  |

**Table S8.** Genetic correlation matrices for each experimental group. Numbers in parentheses are 1 SEM. **Bold** font indicates estimates >2 SEM above or below zero, approximating statistical significance. Only between-trait correlations were used in corresponding matrix comparisons.

| Genetic correlation matrix: **Control Females** | | | | | |  | Genetic correlation matrix: **Control Males** | | | | |
| --- | --- | --- | --- | --- | --- | --- | --- | --- | --- | --- | --- |
|  | Ln SVL | Ln Area | Hue | Bright | Sat |  | Ln SVL | Ln Area | Hue | Bright | Sat |
| Ln SVL | **—** | 0.371  (0.285) | 0.342  (0.262) | 0.115  (0.318) | 0.479  (0.256) |  | **—** | **0.629**  **(0.270)** | -0.130  (0.287) | -0.320  (0.303) | 0.026  (0.295) |
| Ln Area | 0.371  (0.285) | **—** | -0.096  (0.270) | -0.192  (0.304) | **0.517**  **(0.229)** |  | **0.629**  **(0.270)** | **—** | -0.588  (0.348) | -0.219  (0.388) | 0.295  (0.379) |
| Hue | 0.342  (0.262) | -0.096  (0.270) | **—** | **0.933**  **(0.072)** | -0.218  (0.214) |  | -0.130  (0.287) | -0.588  (0.348) | **—** | 0.170  (0.249) | **-0.585**  **(0.159)** |
| Bright | 0.115  (0.318) | -0.192  (0.304) | **0.933**  **(0.072)** | **—** | **-0.494**  **(0.235)** |  | -0.320  (0.303) | -0.219  (0.388) | 0.170  (0.249) | **—** | **0.667**  **(0.115)** |
| Sat | 0.479  (0.256) | **0.517**  **(0.229)** | -0.218  (0.214) | **-0.494**  **(0.235)** | **—** |  | 0.026  (0.295) | 0.295  (0.379) | **-0.585**  **(0.159)** | **0.667**  **(0.115)** | **—** |
|  |  |  |  |  |  |  |  |  |  |  |  |
| Genetic correlation matrix: **Testosterone Females** | | | | | |  | Genetic correlation matrix: **Testosterone Males** | | | | |
| Ln SVL | **—** | 0.431  (0.316) | -0.180  (0.317) | **-0.539**  **(0.240)** | -0.582  (0.299) |  | **—** | 0.134  (0.430) | -0.547  (0.360) | -0.347  (0.455) | 0.422  (0.469) |
| Ln Area | 0.431  (0.316) | **—** | -0.377  (0.245) | **-0.874**  **(0.126)** | -0.131  (0.273) |  | 0.134  (0.430) | **—** | **-0.762**  **(0.218)** | **-0.731**  **(0.241)** | -0.046  (0.347) |
| Hue | -0.180  (0.317) | -0.377  (0.245) | **—** | **0.442**  **(0.184)** | **-0.492**  **(0.195)** |  | -0.547  (0.360) | **-0.762**  **(0.218)** | **—** | **0.703**  **(0.139)** | -0.385  (0.208) |
| Bright | **-0.539**  **(0.240)** | **-0.874**  **(0.126)** | **0.442**  **(0.184)** | **—** | 0.375  (0.242) |  | -0.347  (0.377) | **-0.731**  **(0.241)** | **0.703**  **(0.139)** | **—** | 0.313  (0.264) |
| Sat | -0.582  (0.299) | -0.131  (0.273) | **-0.492**  **(0.195)** | 0.375  (0.242) | **—** |  | 0.422  (0.469) | -0.046  (0.347) | -0.385  (0.208) | 0.313  (0.264) | **—** |
|  |  |  |  |  |  |  |  |  |  |  |  |

**Table S9.** Phenotypic variance-covariance (**P**) and correlation matrices for each experimental group. Elements on the diagonal are estimates of phenotypic variance, those below the diagonal are phenotypic covariances, and those above the diagonal are phenotypic correlations. Numbers in parentheses are 1 SEM. **Bold** font indicates estimates >2 SEM above or below zero, approximating statistical significance.

| **P** and phenotypic correlation matrix: **Control Females** | | | | | |  | **P** and phenotypic correlation matrix: **Control Males** | | | | |
| --- | --- | --- | --- | --- | --- | --- | --- | --- | --- | --- | --- |
|  | Ln SVL | Ln Area | Hue | Bright | Sat |  | Ln SVL | Ln Area | Hue | Bright | Sat |
| Ln SVL | **0.00154**  **(0.00022)** | **0.425**  **(0.078)** | -0.026  (0.092) | -0.242  (0.142) | -0.014  (0.096) |  | **0.00317**  **(0.00078)** | **0.796**  **(0.051)** | 0.085  (0.089) | **-0.422**  **(0.095)** | **-0.181**  **(0.080)** |
| Ln Area | **0.00393**  **(0.00101)** | **0.05560**  **(0.00745)** | -0.084  (0.085) | -0.011  (0.118) | 0.134  (0.091) |  | **0.0174**  **(0.00512)** | **0.15048**  **(0.03585)** | 0.033  (0.088) | **-0.336**  **(0.103)** | -0.114  (0.082) |
| Hue | -0.00412  (0.01485) | -0.08117  (0.08290) | **16.635**  **(1.958)** | **0.400**  **(0.079)** | 0.085  (0.085) |  | 0.01633  (0.01693) | 0.04362  (0.11522) | **11.549**  **(1.2008)** | 0.091  (0.073) | **-0.375**  **(0.062)** |
| Bright | -0.09392  (0.04818) | -0.02509  (0.27573) | **16.189**  **(4.1671)** | **98.467**  **(18.045)** | -0.048  (0.107) |  | **-0.19739**  **(0.06943)** | **-1.0853**  **(0.4560)** | 2.573  (2.083) | **69.173**  **(9.238)** | **0.419**  **(0.056)** |
| Sat | -0.00503  (0.03382) | 0.28360  (0.19401) | 3.1114  (3.1966) | -4.2905  (9.4633) | **81.041**  **(9.529)** |  | **-0.10678**  **(0.05173)** | -0.46382  (0.34880) | **-13.340**  **(2.791)** | **36.421**  **(6.788)** | **109.322**  **(11.205)** |
|  |  |  |  |  |  |  |  |  |  |  |  |
| **P** and phenotypic correlation matrix: **Testosterone Females** | | | | | |  | **P** and phenotypic correlation matrix: **Testosterone Males** | | | | |
| Ln SVL | **0.00278**  **(0.00052)** | **0.343**  **(0.086)** | 0.097  (0.087) | **-0.372**  **(0.082)** | -0.226  (0.121) |  | **0.00392**  **(0.00062)** | **0.450**  **(0.078)** | 0.061  (0.086) | **-0.323**  **(0.083)** | **-0.386**  **(0.077)** |
| Ln Area | **0.00642**  **(0.00169)** | **0.12576**  **(0.01285)** | -0.036  (0.072) | **-0.342**  **(0.070)** | **0.324**  **(0.074)** |  | **0.00775**  **(0.00220)** | **0.07575**  **(0.01031)** | -0.077  (0.079) | **-0.287**  **(0.080)** | **-0.195**  **(0.086)** |
| Hue | 0.01566  (0.01480) | -0.03956  (0.07946) | **9.404**  **(1.058)** | **0.241**  **(0.078)** | **-0.215**  **(0.080)** |  | 0.01166  (0.01625) | -0.06476  (0.06908) | **9.457**  **(1.013)** | **0.311**  **(0.068)** | **-0.181**  **(0.075)** |
| Bright | **-0.19073**  **(0.06211)** | **-1.1781**  **(0.2785)** | **7.176**  **(2.385)** | **94.429**  **(10.921)** | 0.185  (0.094) |  | **-0.16522**  **(0.05486)** | **-0.64647**  **(0.21575)** | **7.808**  **(2.093)** | **66.768**  **(8.013)** | **0.412**  **(0.068)** |
| Sat | -0.14505  (0.09215) | **1.3974**  **(0.4245)** | **-8.029**  **(3.472)** | 21.855  (12.468) | **147.890**  **(26.740)** |  | **-0.22977**  **(0.06686)** | -0.50994  (0.25993) | **-5.293**  **(2.220)** | **32.002**  **(7.072)** | **90.339**  **(10.479)** |
|  |  |  |  |  |  |  |  |  |  |  |  |

**Table S10.** Summary of tests for differences in **G** and genetic correlation matrices across four experimental groups. The top two sections report random skewers (RS) comparisons using (A) variance standardized **G** (Table S6; Fig. 2) or (B) unstandardized **G** (Table S7). The bottom two sections report comparisons of genetic correlation matrices (Table S8) using (C) Mantel matrix correlations, or (D) a modified version of the T method (see Supplemental Methods). **Bold** values on the diagonal are modes (lower 5% bounds) of the distribution of mean vector or matrix correlations (*r*) or of mean differences in genetic correlations (Δ*r*) between the best estimate of a matrix and each of the 10,000 simulated matrices in its error distribution. Values off the diagonal report *r* and Δ*r* calculated between groups, with *P*-values corresponding to the 5% bound of the null distribution in the same column. * Indicates a significant difference between matrices.

|  | **Control**  **Female** | |  | **Testosterone Female** | |  | **Control**  **Male** | |  | **Testosterone**  **Male** | |
| --- | --- | --- | --- | --- | --- | --- | --- | --- | --- | --- | --- |
| **A. Standardized G (RS)** | ***r*** | ***P* (5%)** |  | ***r*** | ***P* (5%)** |  | ***r*** | ***P* (5%)** |  | ***r*** | ***P* (5%)** |
|  |  |  |  |  |  |  |  |  |  |  |  |
| **Control Female** | **0.938** | **(0.803)** |  | 0.629 | 0.002* |  | 0.673 | 0.004* |  | 0.640 | 0.003* |
| **Testosterone Female** | 0.629 | < 0.001* |  | **0.940** | **(0.778)** |  | 0.845 | 0.124 |  | 0.863 | 0.233 |
| **Control Male** | 0.672 | 0.003* |  | 0.845 | 0.149 |  | **0.935** | **(0.800)** |  | 0.827 | 0.124 |
| **Testosterone Male** | 0.640 | 0.001* |  | 0.863 | 0.202 |  | 0.827 | 0.087 |  | **0.936** | **(0.778)** |
|  |  |  |  |  |  |  |  |  |  |  |  |
| **B. Unstandardized G (RS)** | ***r*** | ***P* (5%)** |  | ***r*** | ***P* (5%)** |  | ***r*** | ***P* (5%)** |  | ***r*** | ***P* (5%)** |
|  |  |  |  |  |  |  |  |  |  |  |  |
| **Control Female** | **0.983** | **(0.862)** |  | 0.729 | 0.016* |  | 0.661 | 0.006* |  | 0.726 | 0.015* |
| **Testosterone Female** | 0.729 | 0.006* |  | **0.984** | **(0.825)** |  | 0.927 | 0.200 |  | 0.979 | 0.672 |
| **Control Male** | 0.661 | 0.002* |  | 0.927 | 0.238 |  | **0.983** | **(0.851)** |  | 0.863 | 0.078 |
| **Testosterone Male** | 0.726 | 0.005* |  | 0.979 | 0.689 |  | 0.863 | 0.059 |  | **0.984** | **(0.834)** |
|  |  |  |  |  |  |  |  |  |  |  |  |
| **C. Correlation (Mantel)** | ***r*** | ***P* (5%)** |  | ***r*** | ***P* (5%)** |  | ***r*** | ***P* (5%)** |  | ***r*** | ***P* (5%)** |
|  |  |  |  |  |  |  |  |  |  |  |  |
| **Control Female** | **0.948** | **(0.564)** |  | 0.312 | 0.028* |  | 0.232 | 0.010* |  | 0.518 | 0.049* |
| **Testosterone Female** | 0.312 | 0.016* |  | **0.946** | **(0.437)** |  | 0.770 | 0.313 |  | 0.657 | 0.118 |
| **Control Male** | 0.232 | 0.011* |  | 0.770 | 0.219 |  | **0.928** | **(0.454)** |  | 0.685 | 0.140 |
| **Testosterone Male** | 0.518 | 0.039* |  | 0.657 | 0.124 |  | 0.685 | 0.195 |  | **0.935** | **(0.52q)** |
|  |  |  |  |  |  |  |  |  |  |  |  |
| **D. Correlation (Modified T)** | **Δ*r*** | ***P* (5%)** |  | **Δ*r*** | ***P* (5%)** |  | **Δ*r*** | ***P* (5%)** |  | **Δ*r*** | ***P* (5%)** |
|  |  |  |  |  |  |  |  |  |  |  |  |
| **Control Female** | **0.161** | **(0.368)** |  | 0.554 | 0.007* 0.01* |  | 0.466 | 0.009* |  | 0.461 | 0.009* |
| **Testosterone Female** | 0.554 | < 0.001* |  | **0.150** | **(0.408)** |  | 0.295 | 0.145 |  | 0.290 | 0.232 |
| **Control Male** | 0.466 | 0.009* |  | 0.295 | 0.146 |  | **0.198** | **(0.418)** |  | 0.347 | 0.116 |
| **Testosterone Male** | 0.461 | 0.009* |  | 0.290 | 0.156 |  | 0.347 | 0.070 |  | **0.192** | **(0.408)** |
|  |  |  |  |  |  |  |  |  |  |  |  |

**Table S11.** Summary of tests for differences in **P** and phenotypic correlation matrices across four experimental groups. The top section (A) reports random skewers (RS) comparisons using **P** matrices (on and below the diagonal in Table S9). The bottom two sections report comparisons of phenotypic correlation matrices (above the diagonal in Table S9) using (B) Mantel matrix correlations, or (C) a modified version of the T method (see Supplemental Methods). **Bold** values on the diagonal are modes (lower 5% bounds) of the distribution of mean vector or matrix correlations (*r*) or of mean differences in genetic correlations (Δ*r*) between the best estimate of a matrix and each of the 10,000 simulated matrices in its error distribution. Values off the diagonal report *r* and Δ*r* calculated between groups, with *P*-values corresponding to the 5% bound of the null distribution in the same column. * Indicates a significant difference between matrices.

|  | **Control**  **Female** | |  | **Testosterone Female** | |  | **Control**  **Male** | |  | **Testosterone**  **Male** | |
| --- | --- | --- | --- | --- | --- | --- | --- | --- | --- | --- | --- |
| **A. P matrix (RS)** | ***r*** | ***P* (5%)** |  | ***r*** | ***P* (5%)** |  | ***r*** | ***P* (5%)** |  | ***r*** | ***P* (5%)** |
|  |  |  |  |  |  |  |  |  |  |  |  |
| **Control Female** | **0.999** | **(0.974)** |  | 0.941 | 0.002* |  | 0.885 | < 0.001* |  | 0.915 | < 0.001* |
| **Testosterone Female** | 0.941 | < 0.004* |  | **0.999** | **(0.977)** |  | 0.974 | 0.004* |  | 0.979 | 0.016* |
| **Control Male** | 0.885 | < 0.001* |  | 0.974 | < 0.036* |  | **0.999** | **(0.987)** |  | 0.990 | 0.159 |
| **Testosterone Male** | 0.915 | < 0.001* |  | 0.979 | 0.060 |  | 0.990 | 0.120 |  | **0.999** | **(0.985)** |
|  |  |  |  |  |  |  |  |  |  |  |  |
| **B. Correlation (Mantel)** | ***r*** | ***P* (5%)** |  | ***r*** | ***P* (5%)** |  | ***r*** | ***P* (5%)** |  | ***r*** | ***P* (5%)** |
|  |  |  |  |  |  |  |  |  |  |  |  |
| **Control Female** | **0.943** | **(0.719)** |  | 0.685 | 0.004* |  | 0.560 | < 0.001* |  | 0.614 | < 0.001* |
| **Testosterone Female** | 0.685 | 0.036* |  | **0.957** | **(0.852)** |  | 0.779 | 0.003* |  | 0.778 | 0.005* |
| **Control Male** | 0.560 | 0.011* |  | 0.779 | 0.014* |  | **0.990** | **(0.929)** |  | 0.891 | 0.038* |
| **Testosterone Male** | 0.614 | 0.018* |  | 0.778 | 0.013* |  | 0.891 | 0.015* |  | **0.981** | **(0.904)** |
|  |  |  |  |  |  |  |  |  |  |  |  |
| **C. Correlation (Modified T)** | **Δ*r*** | ***P* (5%)** |  | **Δ*r*** | ***P* (5%)** |  | **Δ*r*** | ***P* (5%)** |  | **Δ*r*** | ***P* (5%)** |
|  |  |  |  |  |  |  |  |  |  |  |  |
| **Control Female** | **0.083** | **(0.141)** |  | 0.182 | 0.005* |  | 0.273 | < 0.001* |  | 0.193 | 0.002* |
| **Testosterone Female** | 0.182 | 0.006* |  | **0.068** | **(0.131)** |  | 0.163 | 0.009* |  | 0.130 | 0.027* |
| **Control Male** | 0.273 | < 0.001* |  | 0.163 | 0.011* |  | **0.059** | **(0.124)** |  | 0.133 | 0.023* |
| **Testosterone Male** | 0.193 | 0.003* |  | 0.130 | 0.053 |  | 0.133 | 0.035* |  | **0.068** | **(0.117)** |
|  |  |  |  |  |  |  |  |  |  |  |  |

**Table S12.** Summary of model comparisons testing for significant additive between-sex genetic covariance in three estimates of the full **G** matrix (including **B**) for different combinations of female and male treatment groups. In the two combinations that included females treated with testosterone, models that included between-sex genetic covariance (i.e., the full **G** matrix plus **B**) were preferred over simpler models that set the between-sex covariances in **B** = 0, indicating significant between-sex genetic covariance. However, in the “natural” **G** matrix estimated for control females and control males, the addition of **B** did not significantly improve model fit, indicating relatively weak between-sex genetic covariance. Chi-squared values are calculated as twice the difference in log likelihood between models and are shown for each of the reduced models in comparison to the preferred full **G** model.

| **Experimental groups** | **Matrix** | **Log Likelihood** | **df** | ***χ ^2^*** | ***P*** |
| --- | --- | --- | --- | --- | --- |
|  |  |  |  |  |  |
| Control Female + Control Male | Full **G** + **B** | –5551.21 | — | — | — |
|  | Only **G**, **B** = 0 | –5567.28 | 25 | 32.62 | 0.141 |
|  |  |  |  |  |  |
| Testosterone Female + Control Male | Full **G** + **B** | –5887.73 | — | — | — |
|  | Only **G**, **B** = 0 | –5913.98 | 25 | 52.49 | 0.001* |
|  |  |  |  |  |  |
| Testosterone Female + Testosterone Male | Full **G** + **B** | –5979.57 | — | — | — |
|  | Only **G**, **B** = 0 | –5999.89 | 25 | 40.65 | 0.025* |
|  |  |  |  |  |  |

**Table S13.** Full variance-standardized genetic variance-covariance matrices (**G**), including the between-sex covariance matrix (**B**), for three combinations of male and female treatments. The upper right quadrat reports corresponding between-sex genetic correlations (*r*_MF_). **Bold** font indicates estimates >2 SEM above or below zero, approximating statistical significance.

| **G** and **B** for **control males** and **control females** with between-sex genetic correlations above diagonal in box | | | | | | | | | | |
| --- | --- | --- | --- | --- | --- | --- | --- | --- | --- | --- |
|  | Ln SVL _M_ | Ln Area _M_ | Hue _M_ | Bright _M_ | Sat _M_ | Ln SVL _F_ | Ln Area _F_ | Hue _F_ | Bright _F_ | Sat _F_ |
| Ln SVL _M_ | **0.141**  **(0.066)** |  |  |  |  | 0.443  (0.263) | **0.582**  **(0.256)** | -0.001  (0.248) | -0.190  (0.281) | 0.255  (0.257) |
| Ln Area _M_ | 0.065  (0.047) | 0.083  (0.051) |  |  |  | 0.269  (0.316) | 0.260  (0.318) | -0.339  (0.282) | **-0.630**  **(0.226)** | 0.212  (0.300) |
| Hue _M_ | -0.070  (0.075) | **-0.142**  **(0.069)** | **0.634**  **(0.166)** |  |  | 0.067  (0.218) | -0.237  (0.224) | **0.856**  **(0.095)** | **0.856**  **(0.126)** | -0.214  (0.206) |
| Bright _M_ | -0.060  (0.057) | -0.033  (0.048) | 0.081  (0.092) | **0.229**  **(0.093)** |  | -0.125  (0.289) | -0.255  (0.270) | -0.032  (0.217) | 0.200  (0.241) | -0.029  (0.264) |
| Sat _M_ | 0.023  (0.070) | 0.065  (0.062) | **-0.313**  **(0.124)** | 0.185  (0.094) | **0.437**  **(0.156)** | 0.169  (0.267) | 0.202  (0.258) | **-0.573**  **(0.166)** | **-0.477**  **(0.225)** | 0.350  (0.233) |
| Ln SVL _F_ | 0.084  (0.058) | 0.039  (0.050) | 0.027  (0.088) | -0.030  (0.073) | 0.057  (0.092) | **0.256**  **(0.105)** |  |  |  |  |
| Ln Area _F_ | 0.125  (0.067) | 0.043  (0.054) | -0.108  (0.104) | -0.070  (0.080) | 0.076  (0.101) | 0.119  (0.087) | **0.327**  **(0.134)** |  |  |  |
| Hue _F_ | -0.001  (0.076) | -0.080  (0.069) | **0.557**  **(0.146)** | -0.013  (0.085) | **-0.309**  **(0.119)** | 0.173  (0.095) | -0.062  (0.109) | **0.668**  **(0.190)** |  |  |
| Bright _F_ | -0.026  (0.041) | -0.066  (0.041) | **0.248**  **(0.072)** | 0.035  (0.044) | **-0.115**  **(0.057)** | 0.011  (0.046) | -0.043  (0.053) | **0.249**  **(0.083)** | **0.133**  **(0.050)** |  |
| Sat _F_ | 0.070  (0.074) | 0.045  (0.064) | -0.125  (0.122) | -0.010  (0.093) | 0.170  (0.120) | 0.199  (0.100) | **0.239**  **(0.115)** | -0.102  (0.127) | -0.130  (0.070) | **0.538**  **(0.181)** |
|  |  |  |  |  |  |  |  |  |  |  |

| **G** and **B** for **control males** and **testosterone females** with between-sex genetic correlations above diagonal in box | | | | | | | | | | |
| --- | --- | --- | --- | --- | --- | --- | --- | --- | --- | --- |
|  | Ln SVL _M_ | Ln Area _M_ | Hue _M_ | Bright _M_ | Sat _M_ | Ln SVL _F_ | Ln Area _F_ | Hue _F_ | Bright _F_ | Sat _F_ |
| Ln SVL _M_ | **0.163**  **(0.066)** |  |  |  |  | **0.790**  **(0.130)** | **0.653**  **(0.168)** | -0.389  (0.222) | **-0.581**  **(0.177)** | 0.045  (0.234) |
| Ln Area _M_ | 0.073  (0.040) | **0.097**  **(0.039)** |  |  |  | 0.202  (0.255) | **0.659**  **(0.157)** | **-0.594**  **(0.172)** | -0.282  (0.215) | **0.663**  **(0.148)** |
| Hue _M_ | -0.086  (0.072) | **-0.141**  **(0.059)** | **0.628**  **(0.161)** |  |  | -0.303  (0.186) | -0.214  (0.227) | **0.819**  **(0.118)** | 0.251  (0.204) | **-0.435**  **(0.177)** |
| Bright _M_ | -0.063  (0.053) | -0.007  (0.038) | 0.095  (0.088) | **0.239**  **(0.078)** |  | -0.422  (0.220) | **-0.606**  **(0.161)** | 0.146  (0.217) | **0.864**  **(0.108)** | **0.591**  **(0.173)** |
| Sat _M_ | 0.028  (0.062) | 0.080  (0.046) | **-0.332**  **(0.115)** | 0.198  (0.070) | **0.478**  **(0.136)** | 0.105  (0.224) | 0.337  (0.193) | **-0.530**  **(0.161)** | **0.464**  **(0.166)** | **0.734**  **(0.121)** |
| Ln SVL _F_ | **0.124**  **(0.050)** | 0.025  (0.034) | -0.094  (0.064) | -0.080  (0.050) | 0.028  (0.063) | **0.152**  **(0.056)** |  |  |  |  |
| Ln Area _F_ | **0.125**  **(0.060)** | **0.097**  **(0.048)** | -0.080  (0.088) | **-0.140**  **(0.053)** | -0.110  (0.069) | 0.081  (0.056) | **0.224**  **(0.090)** |  |  |  |
| Hue _F_ | -0.120  (0.078) | **-0.142**  **(0.057)** | **0.499**  **(0.135)** | 0.054  (0.083) | **-0.281**  **(0.114)** | -0.052  (0.069) | -0.092  (0.090) | **0.591**  **(0.189)** |  |  |
| Bright _F_ | **-0.128**  **(0.058)** | -0.048  (0.040) | 0.108  (0.091) | **0.230**  **(0.066)** | **0.175**  **(0.079)** | **-0.110**  **(0.055)** | **-0.209**  **(0.072)** | 0.154  (0.101) | **0.296**  **(0.093)** |  |
| Sat _F_ | 0.010  (0.053) | **0.114**  **(0.047)** | **-0.189**  **(0.089)** | **0.159**  **(0.063)** | **0.279**  **(0.079)** | -0.072  (0.053) | -0.021  (0.054) | **-0.226**  **(0.091)** | 0.123  (0.068) | **0.302**  **(0.093)** |
|  |  |  |  |  |  |  |  |  |  |  |

| **G** and **B** for **testosterone males** and **testosterone females** with between-sex genetic correlations above diagonal in box | | | | | | | | | | |
| --- | --- | --- | --- | --- | --- | --- | --- | --- | --- | --- |
|  | Ln SVL _M_ | Ln Area _M_ | Hue _M_ | Bright _M_ | Sat _M_ | Ln SVL _F_ | Ln Area _F_ | Hue _F_ | Bright _F_ | Sat _F_ |
| Ln SVL _M_ | **0.101**  **(0.049)** |  |  |  |  | **0.753**  **(0.135)** | 0.112  (0.300) | -0.258  (0.203) | -0.038  (0.284) | 0.043  (0.267) |
| Ln Area _M_ | 0.051  (0.041) | **0.168**  **(0.072)** |  |  |  | **0.684**  **(0.161)** | **0.611**  **(0.186)** | **-0.663**  **(0.142)** | **-0.779**  **(0.109)** | -0.061  (0.247) |
| Hue _M_ | **-0.166**  **(0.076)** | **-0.230**  **(0.083)** | **0.642**  **(0.180)** |  |  | **-0.451**  **(0.195)** | -0.134  (0.236) | **0.840**  **(0.088)** | 0.293  (0.196) | -0.351  (0.180) |
| Bright _M_ | -0.101  (0.059) | **-0.214**  **(0.084)** | **0.403**  **(0.123)** | **0.451**  **(0.171)** |  | **-0.645**  **(0.166)** | -0.211  (0.255) | **0.491**  **(0.175)** | 0.415  (0.221) | 0.198  (0.215) |
| Sat _M_ | 0.029  (0.040) | -0.015  (0.051) | **-0.199**  **(0.085)** | 0.062  (0.087) | **0.260**  **(0.079)** | -0.363  (0.236) | -0.214  (0.244) | **-0.638**  **(0.153)** | 0.214  (0.222) | **0.844**  **(0.108)** |
| Ln SVL _F_ | 0.086  (0.050) | 0.101  (0.061) | -0.130  (0.074) | **-0.155**  **(0.074)** | -0.067  (0.056) | 0.129  (0.072) |  |  |  |  |
| Ln Area _F_ | 0.018  (0.050) | 0.123  (0.069) | -0.053  (0.095) | -0.069  (0.089) | -0.053  (0.068) | 0.066  (0.061) | 0.240  (0.141) |  |  |  |
| Hue _F_ | -0.062  (0.053 | **-0.205**  **(0.079)** | **0.507**  **(0.143)** | **0.248**  **(0.106)** | **-0.245**  **(0.092)** | -0.033  (0.065) | -0.102  (0.095) | **0.566**  **(0.171)** |  |  |
| Bright _F_ | -0.006  (0.047) | **-0.165**  **(0.068)** | 0.121  (0.091) | 0.144  (0.081) | 0.056  (0.059) | -0.084  (0.057) | **-0.210**  **(0.101)** | 0.162  (0.094) | **0.267**  **(0.103)** |  |
| Sat _F_ | 0.007  (0.044) | -0.013  (0.052) | -0.144  (0.085) | 0.068  (0.079) | **0.221**  **(0.068)** | -0.083  (0.054) | -0.026  (0.064) | **-0.217**  **(0.089)** | 0.082  (0.067) | **0.262**  **(0.095)** |
|  |  |  |  |  |  |  |  |  |  |  |

**Table S14.** Full unstandardized genetic variance-covariance matrices (**G**), including the between-sex covariance matrix (**B**), for three combinations of male and female treatments. The upper right quadrat reports corresponding between-sex genetic correlations (*r*_MF_). **Bold** font indicates estimates >2 SEM above or below zero, approximating statistical significance.

| **G** and **B** for **control males** and **control females** with between-sex genetic correlations above diagonal in box | | | | | | | | | | |
| --- | --- | --- | --- | --- | --- | --- | --- | --- | --- | --- |
|  | Ln SVL _M_ | Ln Area _M_ | Hue _M_ | Bright _M_ | Sat _M_ | Ln SVL _F_ | Ln Area _F_ | Hue _F_ | Bright _F_ | Sat _F_ |
| Ln SVL _M_ | **0.00048**  **(0.00022)** |  |  |  |  | 0.443  (0.263) | **0.582**  **(0.256)** | -0.001  (0.248) | -0.190  (0.281) | 0.255  (0.257) |
| Ln Area _M_ | 0.00150  (0.00108) | 0.01308  (0.00808) |  |  |  | 0.269  (0.316) | 0.260  (0.318) | -0.339  (0.282) | **-0.630**  **(0.226)** | 0.212  (0.300) |
| Hue _M_ | -0.01410  (0.01505) | **-0.19268**  **(0.09450)** | **7.4638**  **(1.9603)** |  |  | 0.067  (0.218) | -0.237  (0.224) | **0.856**  **(0.095)** | **0.856**  **(0.126)** | -0.214  (0.206) |
| Bright _M_ | -0.02956  (0.02812) | -0.01104  (0.16092) | 2.3422  (2.6368) | **16.1486**  **(6.5648)** |  | -0.125  (0.289) | -0.255  (0.270) | -0.032  (0.217) | 0.200  (0.241) | -0.029  (0.264) |
| Sat _M_ | 0.01389  (0.04285) | 0.26991  (0.25569) | **-11.2354**  **(4.4424)** | 16.2746  (8.3152) | **47.9309**  **(17.0944)** | 0.169  (0.267) | 0.202  (0.258) | **-0.573**  **(0.166)** | **-0.477**  **(0.225)** | 0.350  (0.233) |
| Ln SVL _F_ | 0.00004  (0.00014) | 0.00063  (0.00079) | 0.00370  (0.01213) | -0.01022  (0.02458) | 0.02370  (0.03972) | **0.00041**  **(0.00017)** |  |  |  |  |
| Ln Area _F_ | 0.00171  (0.00091) | 0.00400  (0.00504) | -0.08715  (0.08358) | -0.13771  (0.15757) | 0.18751  (0.24898) | 0.00112  (0.00082) | **0.01806**  **(0.00738)** |  |  |  |
| Hue _F_ | -0.00001  (0.01821) | -0.13012  (0.11271) | **7.8539**  **(2.0522)** | -0.43307  (2.94525) | **-13.3216**  **(5.13778)** | 0.02845  (0.01575) | -0.05958  (0.10551) | **11.2918**  **(3.2128)** |  |  |
| Bright _F_ | -0.01530  (0.02410) | -0.26488  (0.16366) | **8.5984**  **(2.5040)** | 2.9513  (3.7458) | **-12.1292**  **(6.02776)** | 0.00456  (0.01843) | -0.10137  (0.12630) | **10.3346**  **(3.4264)** | **13.5126**  **(5.0548)** |  |
| Sat _F_ | 0.03682  (0.03877) | 0.15978  (0.22873) | -3.8454  (3.7739) | -0.77655  (7.03649) | 15.9731  (11.2576) | 0.07169  (0.03623) | **0.50522**  **(0.24476)** | -3.7493  (4.6739) | -11.8242  (6.3608) | **43.4240**  **(14.5926)** |
|  |  |  |  |  |  |  |  |  |  |  |

| **G** and **B** for **control males** and **testosterone females** with between-sex genetic correlations above diagonal in box | | | | | | | | | | |
| --- | --- | --- | --- | --- | --- | --- | --- | --- | --- | --- |
|  | Ln SVL _M_ | Ln Area _M_ | Hue _M_ | Bright _M_ | Sat _M_ | Ln SVL _F_ | Ln Area _F_ | Hue _F_ | Bright _F_ | Sat _F_ |
| Ln SVL _M_ | **0.00053**  **(0.00022)** |  |  |  |  | **0.790**  **(0.130)** | **0.653**  **(0.168)** | -0.389  (0.222) | **-0.581**  **(0.177)** | 0.045  (0.234) |
| Ln Area _M_ | 0.00162  (0.00089) | **0.01442**  **(0.00578)** |  |  |  | 0.202  (0.255) | **0.659**  **(0.157)** | **-0.594**  **(0.172)** | -0.282  (0.215) | **0.663**  **(0.148)** |
| Hue _M_ | -0.01660  (0.01395) | **-0.18534**  **(0.07812)** | **7.24808**  **(1.85761)** |  |  | -0.303  (0.186) | -0.214  (0.227) | **0.819**  **(0.118)** | 0.251  (0.204) | **-0.435**  **(0.177)** |
| Bright _M_ | -0.02950  (0.02840) | -0.02272  (0.11947) | 2.64244  (2.46781) | **16.1271**  **(5.28148)** |  | -0.422  (0.220) | **-0.606**  **(0.161)** | 0.146  (0.217) | **0.864**  **(0.108)** | **0.591**  **(0.173)** |
| Sat _M_ | 0.01640  (0.03705) | 0.32090  (0.18542) | **-11.7235**  **(4.05391)** | **16.9069**  **(6.01738)** | **51.7255**  **(14.7601)** | 0.105  (0.224) | 0.337  (0.193) | **-0.530**  **(0.161)** | **0.464**  **(0.166)** | **0.734**  **(0.121)** |
| Ln SVL _F_ | **0.00038**  **(0.00015)** | 0.00005  (0.00067) | -0.01682  (0.01150) | -0.03489  (0.02159) | 0.01558  (0.03450) | **0.00042**  **(0.00016)** |  |  |  |  |
| Ln Area _F_ | **0.00249**  **(0.00119)** | **0.01310**  **(0.00652)** | -0.09521  (0.10459) | **-0.40269**  **(0.15336)** | -0.40157  (0.25188) | 0.00150  (0.00103) | **0.02739**  **(0.01104)** |  |  |  |
| Hue _F_ | -0.02135  (0.01373) | **-0.16997**  **(0.06855)** | **5.25866**  **(1.42539)** | 1.39781  (2.12301) | **-9.08134**  **(3.66558)** | -0.00849  (0.01127) | -0.09999  (0.09744) | **5.68327**  **(1.81395)** |  |  |
| Bright _F_ | **-0.07072**  **(0.03204)** | -0.17859  (0.15062) | 3.56250  (2.98229) | **18.3098**  **(5.26658)** | **17.6182**  **(8.00705)** | **-0.05657**  **(0.02802)** | **-0.70692**  **(0.24515)** | 4.63125  (3.03561) | **27.8745**  **(8.76905)** |  |
| Sat _F_ | 0.00686  (0.03579) | **0.52140**  **(0.21796)** | **-7.66117**  **(3.59086)** | **15.5311**  **(6.14651)** | **34.5850**  **(9.84248)** | -0.04527  (0.03344) | -0.08807  (0.22415) | **-8.33429**  **(3.36400)** | 14.2488  (7.83097) | **42.8841**  **(13.2088)** |
|  |  |  |  |  |  |  |  |  |  |  |

| **G** and **B** for **testosterone males** and **testosterone females** with between-sex genetic correlations above diagonal in box | | | | | | | | | | |
| --- | --- | --- | --- | --- | --- | --- | --- | --- | --- | --- |
|  | Ln SVL _M_ | Ln Area _M_ | Hue _M_ | Bright _M_ | Sat _M_ | Ln SVL _F_ | Ln Area _F_ | Hue _F_ | Bright _F_ | Sat _F_ |
| Ln SVL _M_ | 0.00036  (0.00020) |  |  |  |  | **0.753**  **(0.135)** | 0.112  (0.300) | -0.258  (0.203) | -0.038  (0.284) | 0.043  (0.267) |
| Ln Area _M_ | 0.00123  (0.00114) | 0.02960  (0.01736) |  |  |  | **0.684**  **(0.161)** | **0.611**  **(0.186)** | **-0.663**  **(0.142)** | -0.779  (0.109) | -0.061  (0.247) |
| Hue _M_ | -0.00544  (0.01059) | -0.11009  (0.10340) | **5.38556**  **(1.62993)** |  |  | **-0.451**  **(0.195)** | -0.134  (0.236) | **0.840**  **(0.088)** | 0.293  (0.196) | -0.351  (0.180) |
| Bright _M_ | -0.04290  (0.02912) | **-0.71213**  **(0.34460)** | 4.84059  (2.80886) | **24.9467**  **(9.59110)** |  | **-0.645**  **(0.166)** | -0.211  (0.255) | **0.491**  **(0.175)** | 0.415  (0.221) | 0.198  (0.215) |
| Sat _M_ | -0.05384  (0.03543) | -0.11253  (0.27769) | **-8.21098**  **(3.36623)** | 9.79475  (7.99413) | **39.5306**  **(14.3405)** | -0.363  (0.236) | -0.214  (0.244) | **-0.638**  **(0.153)** | 0.214  (0.222) | **0.844**  **(0.108)** |
| Ln SVL _F_ | 0.00029  (0.00017) | 0.00147  (0.00088) | -0.02180  (0.01244) | **-0.06748**  **(0.03231)** | -0.03362  (0.02854) | **0.00004**  **(0.00002)** |  |  |  |  |
| Ln Area _F_ | 0.00039  (0.00111) | 0.01187  (0.00667) | -0.05870  (0.10559) | -0.19919  (0.25664) | -0.17879  (0.22592) | 0.00089  (0.00072) | **0.01276**  **(0.00545)** |  |  |  |
| Hue _F_ | -0.01202  (0.01028) | **-0.17367**  **(0.06683)** | **4.95366**  **(1.40104)** | **6.25520**  **(2.67153)** | **-7.19457**  **(2.70243)** | **-0.03316**  **(0.01526)** | **-0.20028**  **(0.07269)** | **6.44983**  **(1.81076)** |  |  |
| Bright _F_ | -0.00383  (0.02853) | **-0.43954**  **(0.18184)** | 3.71783  (2.78919) | 11.3908  (6.46998) | 5.18578  (5.46878) | -0.05224  (0.03045) | **-0.48135**  **(0.18823)** | **10.4659**  **(3.18589)** | **30.1580**  **(11.4498)** |  |
| Sat _F_ | 0.00545  (0.03373) | -0.04313  (0.17417) | -5.60733  (3.30946) | 6.83373  (7.92587) | **25.8011**  **(7.96558)** | 0.01764  (0.02416) | -0.03839  (0.13276) | **-6.00753**  **(2.57387)** | 4.82750  (6.78015) | **23.6177**  **(7.17787)** |
|  |  |  |  |  |  |  |  |  |  |  |

**Table S15.** Summary of tests for effects of testosterone on the magnitude of between-sex genetic correlations (*r*_MF_) for five homologous traits (diagonals in the between-sex genetic correlation matrices in Tables S13-S14). To compare any two matrices, the value of *r*_MF_ in one matrix was subtracted from the value of *r*_MF_ for the same trait in the other matrix, with the direction of subtraction indicated in the first column. The mean difference in *r*_MF_ across all five traits was then calculated. The second column reports this point estimate of the mean difference across five *r*_MF_ values when using the best estimate of each correlation matrix (Tables S13-S14). To test for significant differences in the magnitude of *r*_MF_ of across matrices, the same process was repeated for each of the 10,000 matrices in the simulated error distribution of each matrix, yielding 10,000 estimates of the mean difference in *r*_MF_ averaged across the five traits in the matrix. If the lower 5% of this simulated distribution includes zero, the two matrices do not differ significantly in the average magnitude of *r*_MF_, as seen when comparing the two matrices in which females received testosterone (FT + MT and FT + MC, bottom row of table). However, both of these matrices exhibited significantly higher average values of *r*_MF_ when compared to the “natural” correlation matrix estimated between control females and control males (FC + MC).

| **Between-sex correlation matrices being compared, direction of comparison** | **Mean difference in *r*_MF_ between the best estimates of each correlation matrix** | **Simulated distributions of mean differences in *r*_MF_ from error distributions of each matrix** | | | |
| --- | --- | --- | --- | --- | --- |
|  |  | **Mean** | **Mode** | **Lower 5%** | ***P*** |
|  |  |  |  |  |  |
| (FT + MC) – (FC + MC) | 0.351 | 0.355 | 0.360 | 0.138 | 0.006* |
| (FT + MT) – (FC + MC) | 0.283 | 0.292 | 0.266 | 0.065 | 0.021* |
| (FT + MC) – (FT + MT) | 0.068 | 0.062 | 0.063 | – 0.110 | 0.275 |
|  |  |  |  |  |  |

**Table S16.** Summary of sexually antagonistic skewers comparison of the full **G** matrix (including **B**) for control females and control males versus those estimated for testosterone females and control males or for testosterone females and testosterone males. Comparisons are made using variance-standardized **G** matrices (as in Fig. 3 and Table S13) and unstandardized **G** matrices (Table S14). The bold values on the diagonal report the mean and upper or lower 5% bound of the distribution of mean vector correlations between male and female evolutionary responses, derived from passing 10,000 sexually antagonistic skewers through each of the 10,000 matrices simulated from error associated with the best estimate of **G**. The values above and below the diagonal replot the same mean between-sex vector correlations alongside *P-*values corresponding to the comparison of that estimate with the 5% upper or lower bound of the distribution in the same column. These are one-tailed tests because the a priori hypothesis is that the mean vector correlation for control females and control males should be lower than the mean vector correlation for the other two matrices that include testosterone females.

|  | **Control Female**  **+ Control Male** | |  | **Testosterone Female**  **+ Control Male** | |  | **Testosterone Female**  **+ Testosterone Male** | |
| --- | --- | --- | --- | --- | --- | --- | --- | --- |
| **Standardized G (+B)** | ***r*** | ***P* (upper 5%)** |  | ***r*** | ***P* (lower 5%)** |  | ***r*** | ***P* (lower 5%)** |
|  |  |  |  |  |  |  |  |  |
| **C Female + C Male** | **0.469** | **(0.595)** |  | 0.469 | 0.017* |  | 0.469 | 0.084 |
| **T Female + C Male** | 0.789 | < 0.001* |  | **0.789** | **(0.542)** |  | 0.789 | 0.875 |
| **T Female + T Male** | 0.705 | 0.002* |  | 0.705 | 0.364 |  | **0.705** | **(0.425)** |
|  |  |  |  |  |  |  |  |  |
| **Unstandardized G (+B)** | ***r*** | ***P* (upper 5%)** |  | ***r*** | ***P* (lower 5%)** |  | ***r*** | ***P* (lower 5%)** |
|  |  |  |  |  |  |  |  |  |
| **C Female + C Male** | **0.125** | **(0.531)** |  | 0.125 | 0.002* |  | 0.125 | 0.059 |
| **T Female + C Male** | 0.739 | 0.003* |  | **0.739** | **(0.452)** |  | 0.739 | 0.870 |
| **T Female + T Male** | 0.544 | 0.045* |  | 0.544 | 0.127 |  | **0.544** | **(0.101)** |
|  |  |  |  |  |  |  |  |  |

**Supplemental Figures**

**Figure S1.** Representative images of individuals from each sex and treatment at 8 months of age (5 months post-treatment), illustrating effects of testosterone on dewlap phenotypes.

**
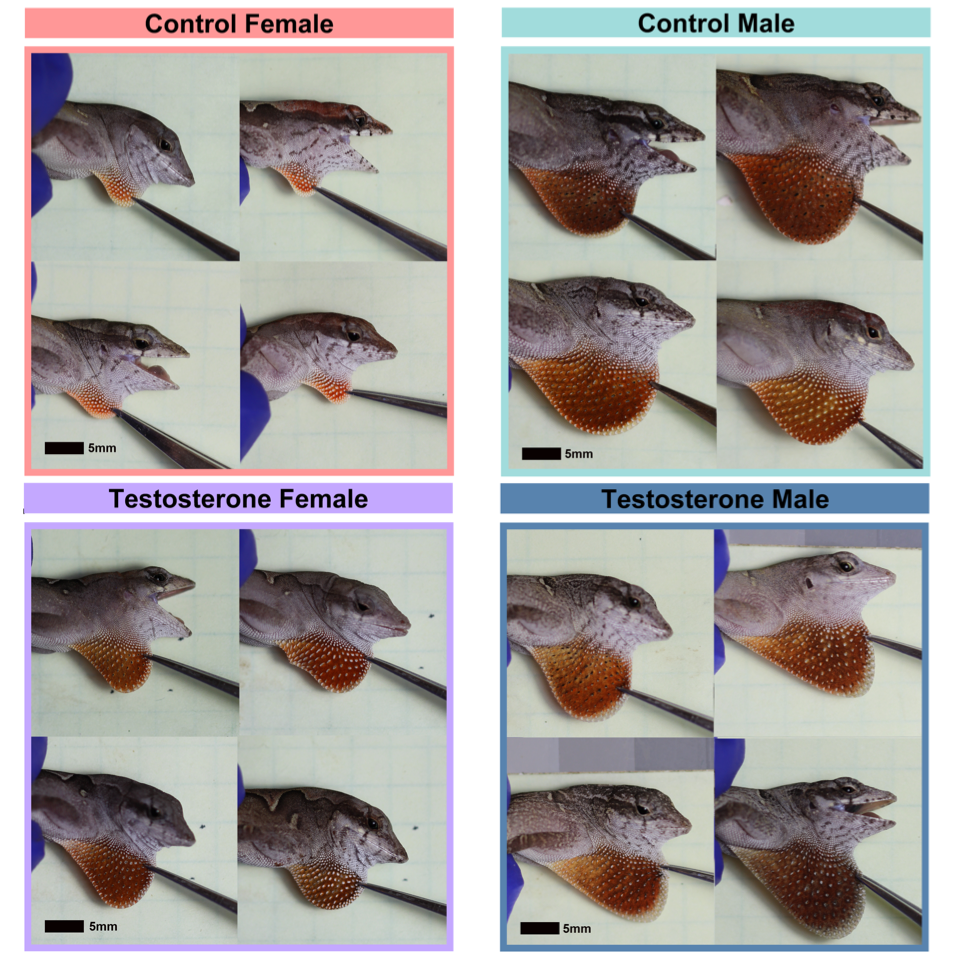
**

**Figure S2.** (A) Separation of experimental groups based on the first two principal components, which explain 71.5% of the variance in 5 phenotypes. Dots represent individuals and ellipses are 95% confidence intervals. For PC1 (positive loading for SVL and dewlap area, negative loading for dewlap brightness), control females are distinct from control and testosterone males, whereas testosterone females are intermediate. For PC2 (positive loading for dewlap saturation, negative loading for dewlap hue), individuals in control groups are slightly left-shifted, whereas individuals in testosterone treatments are slightly right-shifted. (B) Statistical separation of treatment groups on PC1. Solid line = median, box = interquartile range, whiskers = 95% CI, dots = individual outliers. SVL and dewlap area were ln-transformed prior to analysis.

**Figure S3.** Comparisons of within-sex **P** matrices across four experimental groups based on response vectors from random skewers. The null distribution of mean vector correlations between the best estimate of **P** for a group and each of the 10,000 simulated matrices from its own sampling distribution is shown separately for (A) control females, (B) control males, (C) testosterone females, and (D) testosterone males. Dashed lines indicate the lower 5% bound of each distribution. Vertical pins indicate mean vector correlations between the best estimate of **P** for each of three comparison groups to that of the group whose null distribution is shown in that panel. Each vector correlation is plotted on two panels to facilitate comparison to each of the corresponding null distributions.

**
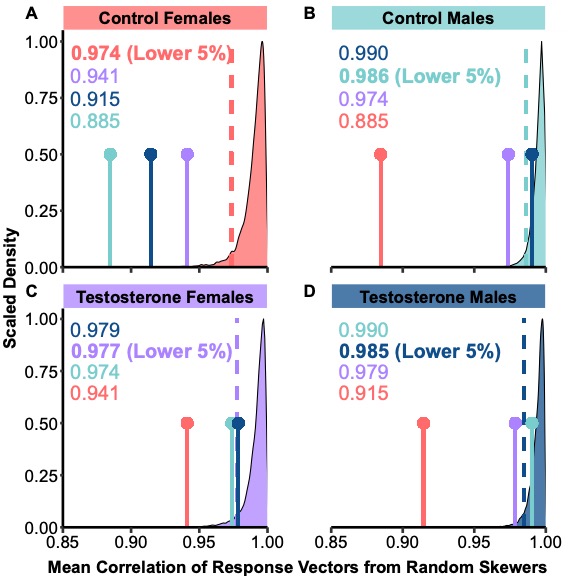
**

**Figure S4.** (A) Point estimates of *r*_MF_ between five homologous traits for control females and control males (FC + MC), connected to the same *r*_MF_ values for testosterone females and testosterone males (FT + MT). Asterisks indicate estimates significantly greater than zero. (B) Distribution of 10,000 mean vector correlations between female and male responses to sexually antagonistic skewers based on the simulated distribution of the full **G** matrix (including **B**) for control females and control males. The upper 5% bound of this null distribution is shown with a dashed line. The mean vector correlation between female and male responses using the best estimate of the full **G** matrix (including **B**) for testosterone females and testosterone males is shown with a pin and falls above the upper 5% bound. (C) The reciprocal comparison to that shown in panel B, with the mean vector correlation for control females and control males falling just within the 95% bound of the simulated distribution for testosterone females and males.

**Figure S5.** Distribution of between-sex selection vector correlations for 10,000 randomly drawn sexually antagonistic skewers (gray distribution), shown alongside corresponding distributions of 10,000 between-sex response vector correlations derived from passing these sexually antagonist skewers through the best estimate of the **G** matrix (including **B**) derived from control females and control males (coral distribution) or from testosterone females and control males (purple distribution). Although both **G** matrices bias the predicted evolutionary response such that it is positively correlated between males and females across the majority of sexually antagonistic selection vectors, this constraint is much stronger when **G** is estimated for testosterone females and males.

**
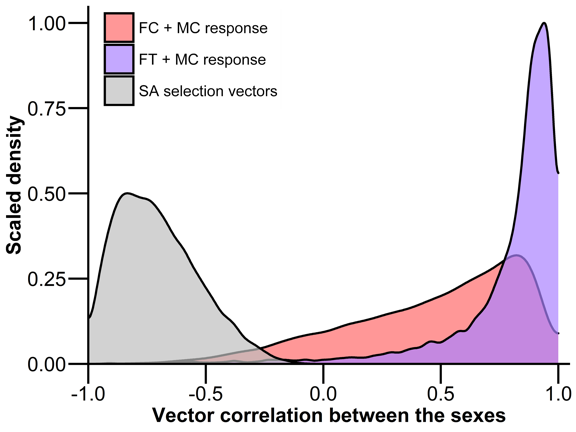
**
